# TGFβ1 Activates Lnc‐APUE to Promote Tumor Metastasis via the *Alu* Element‐Driven STAU1‐Mediated Decay of CDH1 mRNA

**DOI:** 10.1002/advs.202518731

**Published:** 2026-02-03

**Authors:** Song‐Yang Li, Jia‐Hui Huang, Jin‐E Yang, Yi‐Hang Li, Juan‐Zhen Hong, Ting‐Ting Wang, Ying‐Lei Chi, Meng‐Zhi Wu, Wei Wang, Ying Zhu, Shi‐Mei Zhuang

**Affiliations:** ^1^ MOE Key Laboratory of Gene Function and Regulation Guangdong Province Key Laboratory of Pharmaceutical Functional Genes Innovation Center for Evolutionary Synthetic Biology School of Life Sciences State Key Laboratory of Oncology in Southern China Sun Yat‐sen University Guangzhou P. R. China; ^2^ Department of Hepatobiliary Surgery The Second Attached Hospital of Fujian Medical University Quanzhou P. R. China

**Keywords:** CDH1, lnc‐APUE, metastasis, non‐coding RNA, STAU1, TGFβ1

## Abstract

About 25% of long noncoding RNAs (lncRNAs) contain *Alu* elements, yet their functional significance remains largely unexplored. We previously found that lnc‐APUE was upregulated in hepatocellular carcinoma (HCC) and correlated with high recurrence rates. However, the pathogenic roles of lnc‐APUE upregulation in tumor metastasis and its underlying mechanism are still unknown. Here, we showed that an *Alu* element in lnc‐APUE could base‐pair with the *Alu* element in 3’‐untranslated region of E‐cadherin coding gene (CDH1), triggering CDH1 mRNA decay and E‐cadherin loss, consequently enhancing hepatoma cell migration and invasion. These effects of lnc‐APUE were abrogated by deleting or mutating its *Alu* element, or by silencing STAU1 or UPF1, two key components of the STAU1‐mediated mRNA decay (SMD) pathway. Mouse xenograft models revealed that overexpression of wild‐type lnc‐APUE, but not *Alu*‐deleted lnc‐APUE, reduced E‐cadherin levels and promoted tumor metastasis, whereas silencing lnc‐APUE had opposite effects. Furthermore, TGFβ1 stimulation induced SMAD2 binding to the lnc‐APUE promoter, activating its transcription. Silencing lnc‐APUE blocked TGFβ1‐driven migration and invasion, identifying lnc‐APUE as a downstream target and critical mediator of TGFβ1 signaling. Collectively, we define a new TGFβ1/SMAD/lnc‐APUE/E‐cadherin axis: TGFβ1 activates lnc‐APUE to promote cancer metastasis through *Alu* element‐driven STAU1‐mediated CDH1 mRNA decay and subsequent E‐cadherin downregulation.

## Graphical abstract

We delineate a TGFβ1/SMAD/lnc‐APUE/E‐cadherin axis wherein TGFβ1 signaling activates lnc‐APUE; lnc‐APUE subsequently downregulates E‐cadherin expression via *Alu* element‐driven STAU1‐mediated decay of CDH1 mRNA, thereby promoting tumor metastasis. Our findings reveal a novel regulatory function for lnc‐APUE within the SMD pathway, elucidate a distinct mechanism underlying tumor metastasis, and identify a potential therapeutic target for anticancer intervention.

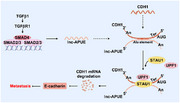

## 1 Introduction

*Alu* elements, a class of highly abundant primate‐specific short interspersed transposable elements (SINEs), constitute more than 10% of the human genome [1, 2, 3]. These ∼280‐nt retrotransposons typically comprise a free left *Alu* monomer (FLAM) and a free right *Alu* monomer (FRAM) joined by a short A‐rich linker [4, 5, 6]. *Alu* elements are preferentially located in gene‐rich genomic regions, particularly in the introns and 3’ untranslated regions (3’UTRs) of mRNAs and in long noncoding RNAs (lncRNAs) [5, 7, 8]. They play critical roles in different physiological and pathological processes [1, 2, 9, 10, 11] by regulating gene expression at the transcriptional, post‐transcriptional, and even translational levels [5, 8]. Around a quarter of lncRNAs carry *Alu* elements [12], yet their regulatory roles remain largely unexplored.

LncRNAs belong to a large family of RNAs longer than 500‐nt without protein‐coding potential [13, 14, 15]. Emerging evidence suggests that lncRNAs participate in diverse biological processes and various diseases by modulating genomic rearrangements, RNA metabolism, and protein function through RNA–DNA, RNA–RNA and RNA–protein interactions [13, 16, 17]. Recent studies have highlighted the regulatory capacity of the *Alu* elements within lncRNAs. For example, the *Alu* element of lncRNA LEADeR interacts with the *Alu* elements in the promoters of interferon‐regulatory factor 1 (IRF1) target genes, which prevents IRF1 from binding to the promoters of its target genes and thus represses target gene transcription, resulting in the inhibition of prostate luminal differentiation [18, 19]. Another study has shown that HNRNPK binds to the *Alu* element in MALAT1 and drives MALAT1 translocation to the nucleus, whereas removal of the *Alu* elements leads to the cytoplasmic accumulation of MALAT1, thus exacerbating DNA damage and apoptosis [19, 20]. It is shown that the *Alu* element within lncRNA ½‐sbsRNA1 can hybridize with the *Alu* element in the 3’UTR of mRNAs to create a STAU1 binding site (SBS). This structure recruits the double‐stranded RNA‐binding protein STAU1, which then engages a key RNA helicase UPF1 to promote SERPINE1 or FLJ21870 mRNA degradation via the STAU1‐mediated mRNA decay (SMD) pathway [11]. Considering the important regulatory roles of *Alu* element and lncRNAs, it is highly worthwhile to identify more *Alu* element‐containing lncRNAs and characterize their function.

Hepatocellular carcinoma (HCC) is a common liver malignancy with poor prognosis due to high metastasis and recurrence rates [21, 22]. Although lncRNAs have been identified as important regulators in HCC development, whether the *Alu* elements within lncRNAs regulate HCC development remains unknown. Transforming growth factor‐beta 1 (TGFβ1) is a fundamental and highly pleiotropic cytokine that is abundant in the tumor microenvironment [23]. Enhanced TGFβ1 signaling promotes tumor metastasis and immunosuppression of HCC [24, 25].

We have demonstrated that lnc‐APUE (lncRNA accelerating proliferation by upregulating E2F1) is upregulated in different cancer types, including HCC, and promotes G1/S phase transition and hepatoma cell growth by working as a miR‐20b sponge and thus upregulating E2F1 expression [26]. High lnc‐APUE level is associated with short recurrence‐free survival of HCC patients [26]. However, whether dysregulation of lnc‐APUE contributes to tumor metastasis remains unreported. In this study, we identified lnc‐APUE as a novel *Alu*‐containing lncRNA transcriptionally activated by the TGFβ1‐SMAD2/3/4 pathway. Lnc‐APUE promoted HCC metastasis by base‐pairing with the CDH1 mRNA 3’UTR through the *Alu* element, forming an SBS to activate the SMD pathway and shorten CDH1 mRNA half‐life. These findings highlight a new regulatory role of lnc‐APUE in SMD and tumor metastasis, and provide a potential therapeutic target for HCC.

## 2 Results

### 2.1 Lnc‐APUE Decreases CDH1 mRNA Levels by Base‐Pairing With the CDH1 3’UTR Through an *Alu* Element

**FIGURE 1:**
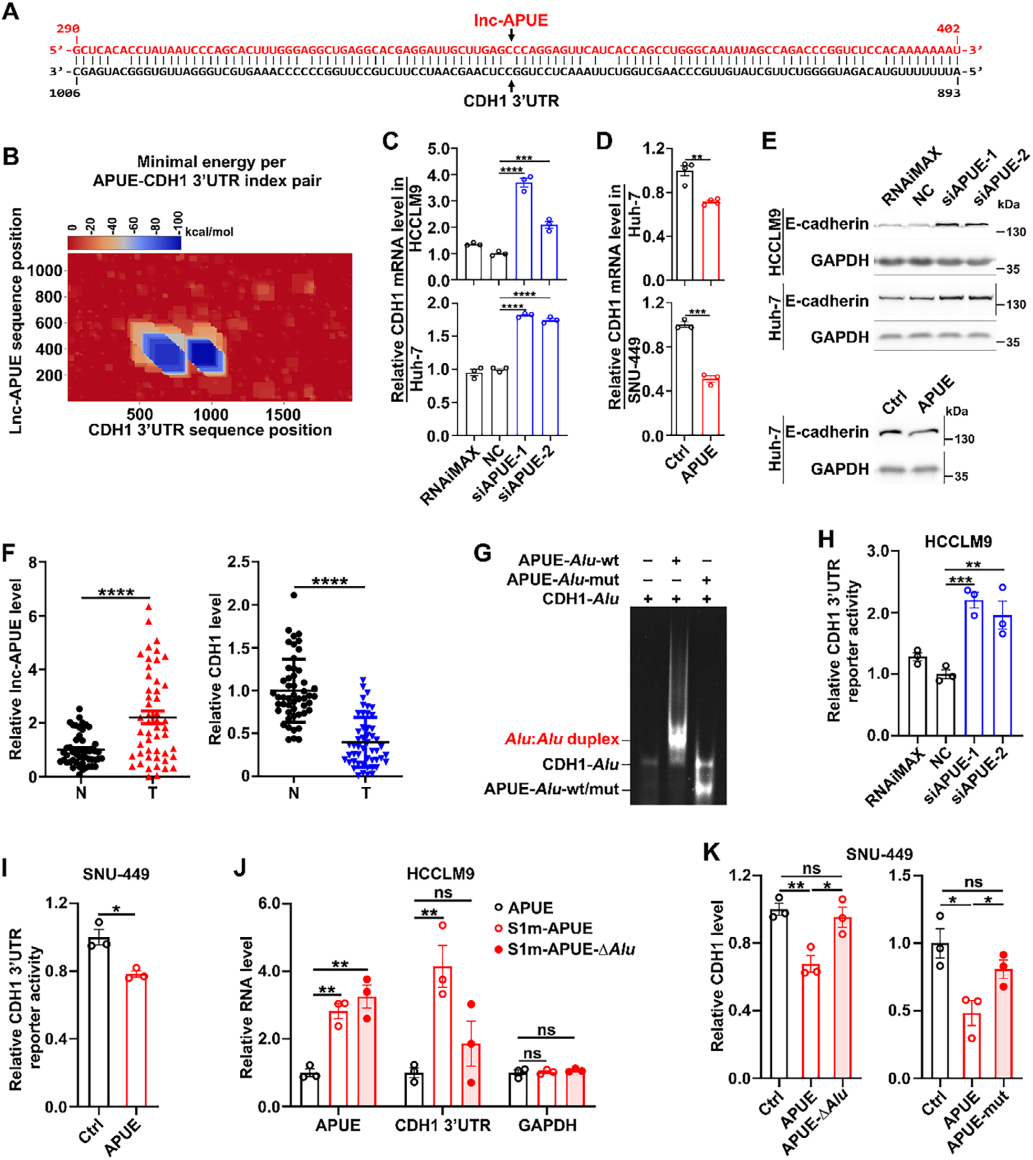
Lnc‐APUE decreases CDH1 mRNA levels by interacting with CDH1 3’UTR via the *Alu* elements. (A) An *Alu* motif within lnc‐APUE base‐paired with a complementary *Alu* element in the CDH1 3’UTR. The interaction between lnc‐APUE and CDH1 was predicted using the NCBI blastn algorithm. (B) The lnc‐APUE *Alu* element and CDH1 3’UTR *Alu* sequence base‐paired with a ΔG of −105.24 kcal/mol. The interaction between lnc‐APUE and CDH1 was predicted using the software IntaRNA. (C) Lnc‐APUE knockdown elevated CDH1 mRNA levels. HCCLM9 and Huh‐7 cells were transfected with the indicated siRNAs for 48 h before qPCR analysis. (D) Ectopic expression of lnc‐APUE downregulated CDH1 transcript abundance. Huh‐APUE and SNU‐APUE stable cell lines and their controls Huh‐Ctrl and SNU‐Ctrl underwent qPCR analysis. (E) The level of E‐cadherin protein was increased by silencing lnc‐APUE but reduced by overexpressing lnc‐APUE. (F) Upregulation of lnc‐APUE and downregulation of CDH1 were frequently observed in human HCC tissues. The mRNA levels of lnc‐APUE and CDH1 were assessed in 50 paired HCC (T) and adjacent non‐tumor liver (N) tissues by qPCR analysis. (G) EMSA revealed a mobility shifted band indicative of the APUE‐*Alu*‐wt:CDH1‐*Alu* RNA duplex formation. APUE‐*Alu*‐wt: wild‐type *Alu* RNA segment (290–402 nt) within lnc‐APUE. APUE‐*Alu* mut: mutant APUE‐*Alu* fragment with complementary nucleotide substitutions. CDH1‐*Alu*: biotin‐labeled *Alu* RNA fragment (893–1006 nt) within the CDH1 3’UTR. “+” or “−” indicates presence (+) or absence (−) of the indicated treatment. (H,I) The luciferase activity of the reporter carrying the CDH1 3’UTR *Alu* element was increased by silencing lnc‐APUE but reduced by overexpressing lnc‐APUE. (J) S1m‐tagged RNA affinity purification assays revealed direct binding of the lnc‐APUE *Alu* element with the CDH1 3’UTR *Alu* element. S1m‐APUE, S1m‐tagged wild‐type lnc‐APUE; S1m‐APUE‐∆*Alu*, S1m‐tagged *Alu*‐deleted lnc‐APUE; APUE, untagged wild‐type lnc‐APUE as a negative control. The indicated RNAs in the S1m‐pulldown precipitates and in the input were assessed by qPCR analysis. The RNA level in the pull‐down product was corrected by that in the input. The mean value of the adjusted RNA level in the pull‐down product of the LM9‐APUE group was set as relative RNA level 1. GAPDH, negative control. (K) Deletion or mutation in the lnc‐APUE *Alu* element abolished the effect of lnc‐APUE in reducing CDH1 mRNA levels. SNU‐APUE, SNU‐APUE‐∆*Alu*, SNU‐APUE‐mut, and their control SNU‐Ctrl were subjected to qPCR analysis. SNU‐APUE‐mut cells harbored complementary nucleotide substitutions within the lnc‐APUE *Alu* sequence. For (B–E,G–K), lnc‐APUE is abbreviated as “APUE”. RNAiMAX, cells exposed to Lipofectamine RNAiMAX without RNA. NC, negative control for siRNA. The data from at least three independent experiments are presented as mean ± SEM (C,D,H–K); *p* values were assessed by one‐way ANOVA (C,H,J,K), unpaired Student′s *t*‐test (D,I), or paired Student′s *t*‐test (F). ^*^, *p* < 0.05; ^**^, *p* < 0.01; ^***^, *p* < 0.001; ^****^, *p* < 0.0001; ns, not significant.

In an attempt to explore the novel function of lnc‐APUE, we identified a single *Alu* element within its sequence (Figure S1A,B). Using IntaRNA and BLASTN, we computationally predicted 1818 candidate mRNAs containing *Alu* element capable of forming complementary base pairs with the *Alu* sequence of lnc‐APUE (Figure S2A). To identify high‐confidence HCC‐relevant SMD substrates among these candidates, we employed the following criteria (Figure S2A): (1) Downregulation in HCC tissues (Fold Change < 0.6) in both GSE77314 and GSE115018 datasets; (2) More than twofold enrichment in STAU1‐bound transcripts in GSE8438. This screening identified five candidate genes, including CDH1, DHODH, TBXA2R, APOL6, and IGF1. Among them, the *Alu* element within the CDH1 3’UTR displayed the highest base‐pairing potential with that of lnc‐APUE (Figure 1A; Figure S2B), with a ΔG value of −105.24 kcal/mol (Figure 1B). While lnc‐APUE silencing upregulated CDH1 mRNA in multiple hepatoma cell lines (Figure 1C; Figure S3A–C), it showed no effect on four other mRNAs (Figure S3D). Analysis of RNA‐sequencing data from 50 paired HCC and adjacent non‐tumor tissues revealed lnc‐APUE expression levels comparable to CDH1 and within the range of well‐characterized oncogenic lncRNAs (Figure S3E). Given this integrated bioinformatic and experimental evidence, we prioritized mechanistic investigation of the lnc‐APUE‐CDH1 interaction.

In contrast to lnc‐APUE silencing (Figure 1C), lnc‐APUE overexpression decreased CDH1 mRNA levels (Figure 1D; Figure S3F,G). Consistently, the protein levels of E‐cadherin, encoded by CDH1 gene, increased following lnc‐APUE silencing but decreased after lnc‐APUE overexpression (Figure 1E). Analysis of clinical samples revealed that compared with noncancerous liver tissues, lnc‐APUE was upregulated and CDH1 was downregulated in HCC tissues (Figure 1F; Figure S3E), suggesting that lnc‐APUE upregulation may reduce CDH1 mRNA levels.

We then investigated whether lnc‐APUE affected CDH1 mRNA levels through the *Alu* element. RNA‐RNA electrophoretic mobility shift assay (EMSA) revealed a mobility‐shifted band upon incubation of the CDH1‐*Alu* segment with wild‐type APUE‐*Alu* fragment, indicating formation of a stable *Alu*:*Alu* heteroduplex. Significantly, the observed mobility shift was eliminated when the APUE‐*Alu* fragment was mutated (APUE‐*Alu* mut) to disrupt its base‐pairing with CDH1‐*Alu* (Figure 1G), indicating that base‐pairing is essential for *Alu*:*Alu* duplex formation. Dual‐luciferase reporter analysis showed that the activity of luciferase carrying CDH1 3’UTR increased after lnc‐APUE knockdown (Figure 1H) but decreased following lnc‐APUE overexpression (Figure 1I), validating that lnc‐APUE downregulates CDH1 expression by binding to CDH1 3’UTR. We then employed S1m‐tag RNA affinity assays to further confirm whether lnc‐APUE directly bound the CDH1 3’UTR through the *Alu* element, using a cell line stably expressing S1m‐tagged wild‐type or *Alu*‐deleted lnc‐APUE or untagged wild‐type lnc‐APUE. Compared to untagged‐lnc‐APUE, both S1m‐APUE and S1m‐APUE‐∆*Alu* precipitates showed significantly enriched lnc‐APUE levels, whereas CDH1 3’UTR enrichment occurred only in S1m‐APUE precipitates and was absent in S1m‐APUE‐∆*Alu* precipitates (Figure 1J). GAPDH, the negative control, was not enriched in any precipitates (Figure 1J). Subsequent mRNA analysis revealed that CDH1 levels decreased after wild‐type lnc‐APUE overexpression, but remained unaffected in cells expressing either *Alu*‐deleted or *Alu*‐mutated lnc‐APUE (Figure 1K). These results indicate that the *Alu* element of lnc‐APUE forms a direct RNA‐RNA duplex with the *Alu* element in the CDH1 3’UTR and this interaction is critical for lnc‐APUE to reduce CDH1 levels.

### 2.2 Lnc‐APUE Promotes CDH1 mRNA Decay Through the *Alu*‐Dependent SMD Pathway

**FIGURE 2:**
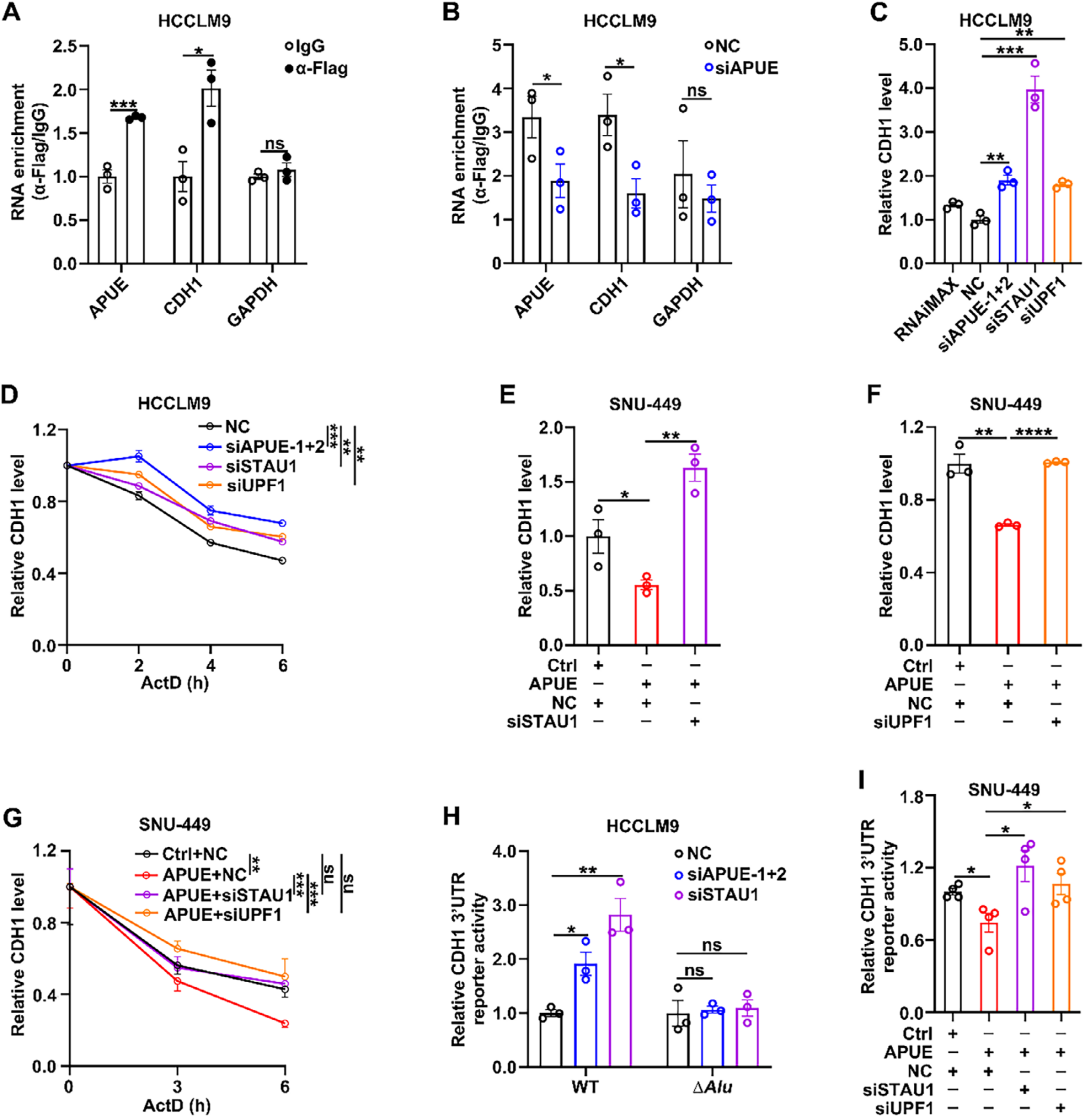
Lnc‐APUE promotes CDH1 mRNA decay via the SMD pathway. (A) STAU1 interacted with lnc‐APUE and CDH1 3’UTR in vivo. RNA immunoprecipitation (RIP) assay was performed in HCCLM9 cells stably expressing STAU1‐Flag using anti‐Flag antibody or isotype‐matched IgG (negative control). Co‐precipitated RNAs were then analyzed by qPCR to detect lnc‐APUE, CDH1 3’UTR, and the negative control transcript GAPDH. (B) Lnc‐APUE knockdown impaired the association of STAU1 with CDH1 3’UTR. LM9‐STAU1‐Flag cells were transfected with the indicated siRNAs for 48 h before RIP assay. (C) Silencing of lnc‐APUE, STAU1, or UPF1 increased CDH1 mRNA levels. HCCLM9 cells were transfected with the indicated siRNAs for 48 h, then subjected to qPCR analysis. (D) Silencing lnc‐APUE, STAU1, or UPF1 extended the half‐life of CDH1 mRNA. (E,F) Silencing STAU1 or UPF1 abolished the effect of lnc‐APUE overexpression in decreasing CDH1 mRNA level. SNU‐Ctrl and SNU‐APUE cells were transfected with NC, siSTAU1 (E), or siUPF1 (F) for 48 h prior to qPCR analysis. (G) Knockdown of either STAU1 or UPF1 abolished lnc‐APUE‐induced degradation of CDH1 mRNA. For (D,G), HCCLM9, SNU‐APUE, and SNU‐Ctrl cells were transfected with the indicated siRNAs for 24 h, then treated with ActD for specified durations, followed by qPCR analysis. (H) Silencing either lnc‐APUE or STAU1 enhanced the luciferase activity of psi‐CDH1‐3’UTR but not that of psi‐CDH1‐3’UTR‐∆*Alu*. HCCLM9 cells were co‐transfected with the indicated siRNAs and the luciferase reporter psi‐CDH1‐3’UTR (WT, wild‐type) or psi‐CDH1‐3’UTR‐∆*Alu* (∆*Alu, Alu*‐deleted) for 48 h, followed by luciferase activity assay. (I) Depletion of STAU1 or UPF1 attenuated the role of lnc‐APUE in repressing the luciferase activity of the psi‐CDH1‐3’UTR reporter. SNU‐APUE and its control SNU‐Ctrl cells were transfected with the siRNAs and psi‐CDH1‐3’UTR reporter for 48 h, then subjected to luciferase activity assay. Lnc‐APUE is abbreviated as “APUE”. RNAiMAX, cells exposed to Lipofectamine RNAiMAX without RNA. NC, negative control for siRNA. The data from at least three independent experiments are presented as mean ± SEM (A–I); *p* values were assessed by unpaired Student′s *t*‐test (A,B), one‐way ANOVA (C,E,F,H,I), or two‐way ANOVA (D,G). ^*^, *p* < 0.05; ^**^, *p* < 0.01; ^***^, *p* < 0.001; ^****^, *p* < 0.0001; ns, not significant.

To assess whether lnc‐APUE downregulated CDH1 mRNA through SMD machinery, hepatoma cells that stably expressed Flag‐tagged STAU1 were applied to RNA immunoprecipitation (RIP) assay. Compared with IgG controls, anti‐Flag immunoprecipitates exhibited significant enrichment of both lnc‐APUE and CDH1 3’UTR, but not GAPDH (Figure 2A), indicating specific in vivo interaction of STAU1 with lnc‐APUE and CDH1 3’UTR. Strikingly, silencing lnc‐APUE diminished STAU1 association with CDH1 3’UTR (Figure 2B), suggesting that lnc‐APUE is required to recruit SMD machinery to the CDH1 transcript. In line with the SMD mechanism, knockdown of key SMD components, STAU1 or UPF1, increased CDH1 mRNA levels (Figure 2C) and prolonged the half‐life of CDH1 mRNA (Figure 2D), which phenocopied the effects of lnc‐APUE depletion (Figure 2C,D; Figure 1C). Importantly, silencing STAU1 or UPF1 abolished the roles of lnc‐APUE in decreasing CDH1 mRNA abundance (Figure 2E,F) and stability (Figure 2G). Consistent with the above results, knockdown of either lnc‐APUE or STAU1 enhanced luciferase activity of the psi‐CDH1‐3’UTR reporter containing wild‐type CDH1 3’UTR, but could not affect the psi‐CDH1‐3’UTR‐∆*Alu* reporter carrying an *Alu*‐deleted CDH1 3’UTR (Figure 2H; Figure S4). Furthermore, depletion of STAU1 or UPF1 attenuated the repressive role of lnc‐APUE on luciferase activity of the psi‐CDH1‐3’UTR reporter (Figure 2I). Collectively, these results imply that lnc‐APUE may downregulate E‐cadherin by promoting SMD‐dependent decay of CDH1 mRNA.

### 2.3 Lnc‐APUE Promotes Tumor Metastasis by Downregulating CDH1 via Its *Alu* Element and STAU1

**FIGURE 3:**
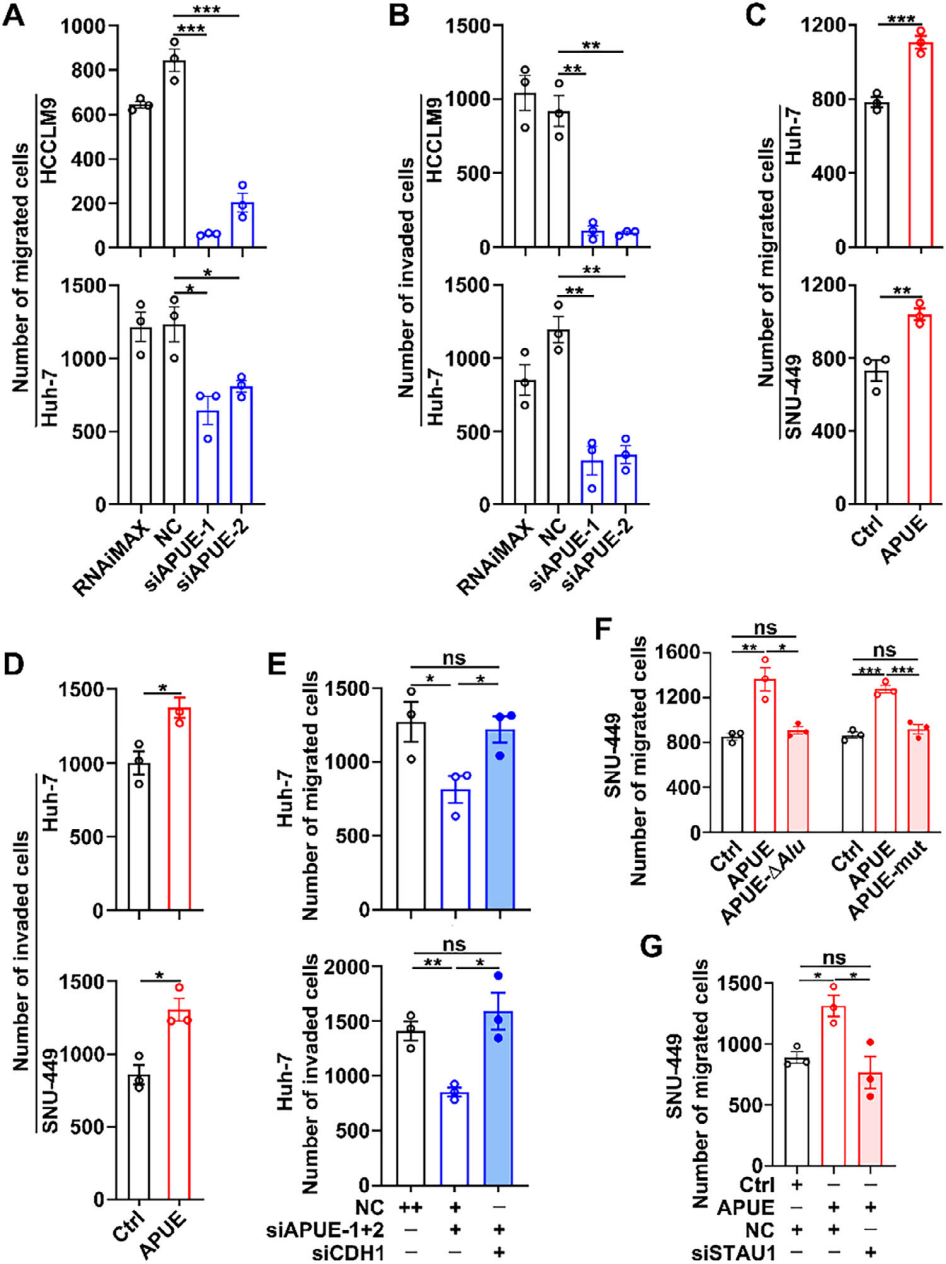
Lnc‐APUE promotes the migration and invasion of human hepatoma cells via STAU1‐mediated CDH1 mRNA decay. (A,B) Silencing lnc‐APUE suppressed the migration and invasion of tumor cells. (C,D) Overexpressing lnc‐APUE promoted the migration and invasion of tumor cells. (E) Silencing CDH1 abolished the inhibitory effects of lnc‐APUE knockdown on tumor cell migration and invasion. (F) Deletion or mutation of the lnc‐APUE *Alu* element abolished the pro‐migratory function of lnc‐APUE in tumor cells. (G) Silencing STAU1 counteracted the pro‐migratory role of lnc‐APUE in tumor cells. For (A,B,E), HCCLM9 and Huh‐7 cells were transfected with the specific siRNAs for 36 h prior to migration and invasion assays. For (C,D,F), Huh‐7 and SNU‐449 cells, with stable transfection of the control (Ctrl), wild‐type (APUE) *Alu*‐deleted (APUE‐∆*Alu*) or *Alu*‐mutated (APUE‐mut) lnc‐APUE constructs, were analyzed. For (G), SNU‐Ctrl and SNU‐APUE were transfected with the indicated siRNAs for 48 h before migration assays. Lnc‐APUE is abbreviated as “APUE”. RNAiMAX, cells exposed to Lipofectamine RNAiMAX without RNA. NC, negative control for siRNA. The data from at least three independent experiments are presented as mean ± SEM (A–G); *p* values were assessed by one‐way ANOVA (A,B,E–G) or unpaired Student′s *t*‐test (C,D). ^*^, *p* < 0.05; ^**^, *p* < 0.01; ^***^, *p* < 0.001; ns, not significant.

Given that E‐cadherin loss drives epithelial to mesenchymal transition and confers migratory/invasive properties to tumor cells [27], the influence of lnc‐APUE/E‐cadherin axis on HCC metastasis was further examined. In vitro transwell assays revealed that the migration and invasion of hepatoma cells were significantly inhibited by silencing lnc‐APUE (Figure 3A,B; Figure S5A,B) but enhanced by overexpressing lnc‐APUE (Figure 3C,D; Figure S5C,D). Moreover, CDH1 silencing abrogated the inhibitory effect of lnc‐APUE knockdown on tumor cell migration and invasion (Figure 3E; Figure S6A), suggesting that lnc‐APUE may promote these malignant phenotypes through decreasing CDH1. Notably, both *Alu*‐deleted and *Alu*‐mutated lnc‐APUE failed to enhance tumor cell migration (Figure 3F; Figure S6B,C). Consistently, STAU1 knockdown abolished the pro‐migratory effect of lnc‐APUE overexpression (Figure 3G; Figure S6D), implying that lnc‐APUE functions via the *Alu* element‐dependent SMD pathway.

**FIGURE 4:**
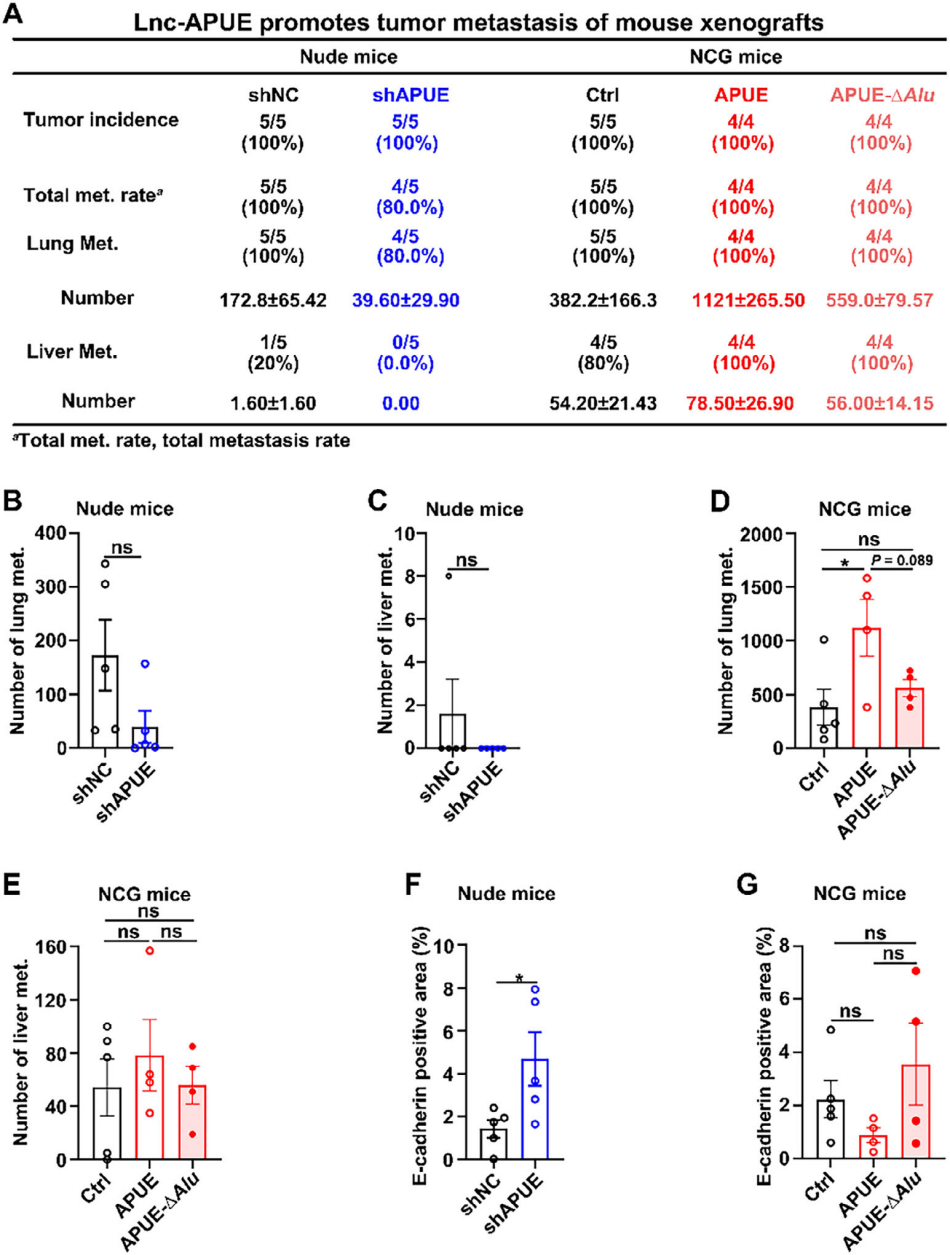
Lnc‐APUE promotes in vivo metastasis of hepatoma cells via the *Alu* element. (A–E) The metastasis of orthotopic liver xenografts was inhibited by silencing lnc‐APUE but was promoted by overexpressing lnc‐APUE in hepatoma cells. For loss‐of‐function, LM9‐shAPUE and its control LM9‐shNC were inoculated under the capsule of the left hepatic lobe of nude mice (*n* = 5 per gheroup). For gain‐of‐function, LM9‐Ctrl (Ctrl, n = 5), LM9‐APUE (APUE, n = 4), and LM9‐APUE‐∆*Alu* (APUE‐∆*Alu*, n = 4) cells were implanted into the liver of NCG mice. Hematoxylin‐eosin staining was performed on 30 serial sections of lung and liver tissues to examine the metastatic nodules. Met., Metastasis. (F) Knockdown of lnc‐APUE elevated E‐cadherin protein levels in mouse xenografts. (G) Wild‐type lnc‐APUE but not *Alu*‐deleted lnc‐APUE reduced E‐cadherin protein levels. For (F,G), E‐cadherin protein in mouse xenografts was determined by immunohistochemical staining. Lnc‐APUE is abbreviated as “APUE”. Data are presented as mean ± SEM (B–G); *p* values were assessed by unpaired Student′s *t*‐test (B,C,F) or one‐way ANOVA (D,E,G). ^*^, *p* < 0.05; ns, not significant.

We next verified the impact of lnc‐APUE on tumor metastasis in vivo, using a mouse orthotopic liver xenograft model. Compared to the control group, xenografts from lnc‐APUE‐silenced hepatoma cells exhibited reductions in primary tumor volume (Figure S7A), metastasis rates, and the number of metastatic nodules in the lungs and livers (Figure 4A–C; Figure S7B). Consistently, compared with the xenografts derived from control cells, tumors from hepatoma cells with stable lnc‐APUE expression showed a larger tumor volume (Figure S8A) and higher numbers of metastatic nodules (Figure 4A,D,E, bar 2 vs. bar 1; Figure S8B); however, deletion of its *Alu* element attenuated these lnc‐APUE‐mediated promotive effects (Figure 4A,D,E, bar 3 vs. bar 1; Figure S8B). Compared with control tumors, E‐cadherin levels were elevated in lnc‐APUE‐silenced xenografts (Figure 4F; Figure S9A) but reduced in lnc‐APUE‐overexpressing tumors (Figure 4G, bar 2 vs. bar 1; Figure S9B). In contrast, tumors with lnc‐APUE‐∆*Alu* overexpression did not exhibit a decrease in E‐cadherin (Figure 4G, bar 3 vs. bar 1; Figure S9B).

Collectively, lnc‐APUE may downregulate CDH1, thereby stimulating hepatoma cell migration, invasion, and metastasis in a manner dependent on its *Alu* element and STAU1.

### 2.4 Lnc‐APUE Transcription Is Induced by TGFβ1/SMAD Signaling

**FIGURE 5:**
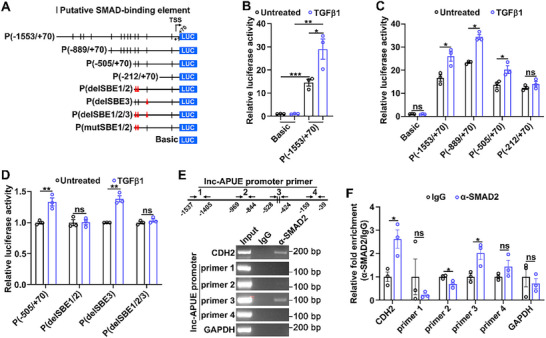
TGFβ1 activates lnc‐APUE transcription. (A) Schematic diagram of *Firefly* luciferase reporters containing the specific DNA fragments of the lnc‐APUE promoter. Arrow denotes the transcription start site (TSS) and transcription direction of lnc‐APUE. Short vertical line, putative SMAD‐binding element (SBE). Red triangle (Δ), deletion of the SBE (delSBE). Red diamond (◊), mutation of the SBE (mutSBE). (B) TGFβ1 treatment enhanced the activity of the lnc‐APUE promoter. (C) The −0.5 to −0.2‐kb region of the lnc‐APUE promoter contained TGFβ1 responsive elements. (D) Deletion of putative SBE1 and SBE2 in the lnc‐APUE promoter abrogated the response of P(−0.5/+0.07k) reporter to TGFβ1. For (B–D), HCCLM9 cells were transfected with the indicated vectors for 36 h. The cells were then either left untreated or treated with TGFβ1 for a further 12 h prior to the luciferase activity assay. (E,F) ChIP analysis showed a direct interaction between SMAD2 and the lnc‐APUE promoter in vivo. ChIP analysis was performed in HCCLM9 cells using anti‐SMAD2 or isotype‐matched IgG, and the antibody‐precipitated DNAs were examined by semi‐quantitative PCR assay (E) or by qPCR analysis (F). Lnc‐APUE promoter amplicons spanning the regions −1537 to −1405‐bp, −969 to −844‐bp, −528 to −424‐bp, and −159 to −39‐bp, were analyzed. The CDH2 and GAPDH promoters were included as positive and negative controls, respectively. The data from at least three independent experiments are presented as mean ± SEM (B–D,F); *p* values were assessed by unpaired Student′s *t*‐test (B–D, F). ^*^, *p* < 0.05; ^**^, *p* < 0.01; ^***^, *p* < 0.001; ns, not significant.

TGFβ1 is highly enriched in the tumor microenvironment, where it activates SMAD signaling to drive metastasis through transcriptional regulation. Thirteen SMAD‐binding elements (SBEs) were predicted within the 1.5‐kb region upstream from the transcriptional start site of lnc‐APUE (Figure 5A). We therefore examined whether lnc‐APUE was a direct transcriptional target of the SMAD complex. Luciferase activity assays revealed that TGFβ1 treatment enhanced the activity of a TGFβ1 responsive SBE reporter, confirming the functional integrity and inducibility of the TGFβ1 signaling pathway in our experimental system (Figure S10A). The promoter reporter construct P(−1553/+70), spanning nucleotides −1553 to +70, showed increased luciferase activity (Figure 5B, bar 3 vs. bar 1), which was further enhanced by TGFβ1 treatment (Figure 5B, bar 4 vs. bar 3). To map the TGFβ1 response element within the lnc‐APUE promoter, sequential 5’‐deletion constructs were generated and functionally assessed. TGFβ1 treatment increased the luciferase activity driven by the −505 to +70‐bp sequence, but had no effect on the reporter containing the −212 to +70‐bp region (Figure 5C), suggesting that the region between −505 and −212‐bp likely harbors TGFβ1‐responsive elements. Deletion or mutation of SBE1/2 within the −505 to −212‐bp region, but not deletion of SBE3, abolished the response of the P(−505/+70) to TGFβ1 (Figure 5A,D; Figure S10B). Subsequent chromatin immunoprecipitation (ChIP) assays showed that compared to IgG controls, anti‐SMAD2 antibody specifically enriched chromatin fragments from the promoter of the known TGFβ1 target gene CDH2 and the putative SMAD‐binding region (−528 to −424‐bp) of the lnc‐APUE promoter by two– to three‐fold, while no enrichment was detected in either the SMAD‐unbound regions of lnc‐APUE or the GAPDH promoter (Figure 5E,F). These findings imply that TGFβ1 stimulation may induce direct SMAD2 binding to the lnc‐APUE promoter.

**FIGURE 6:**
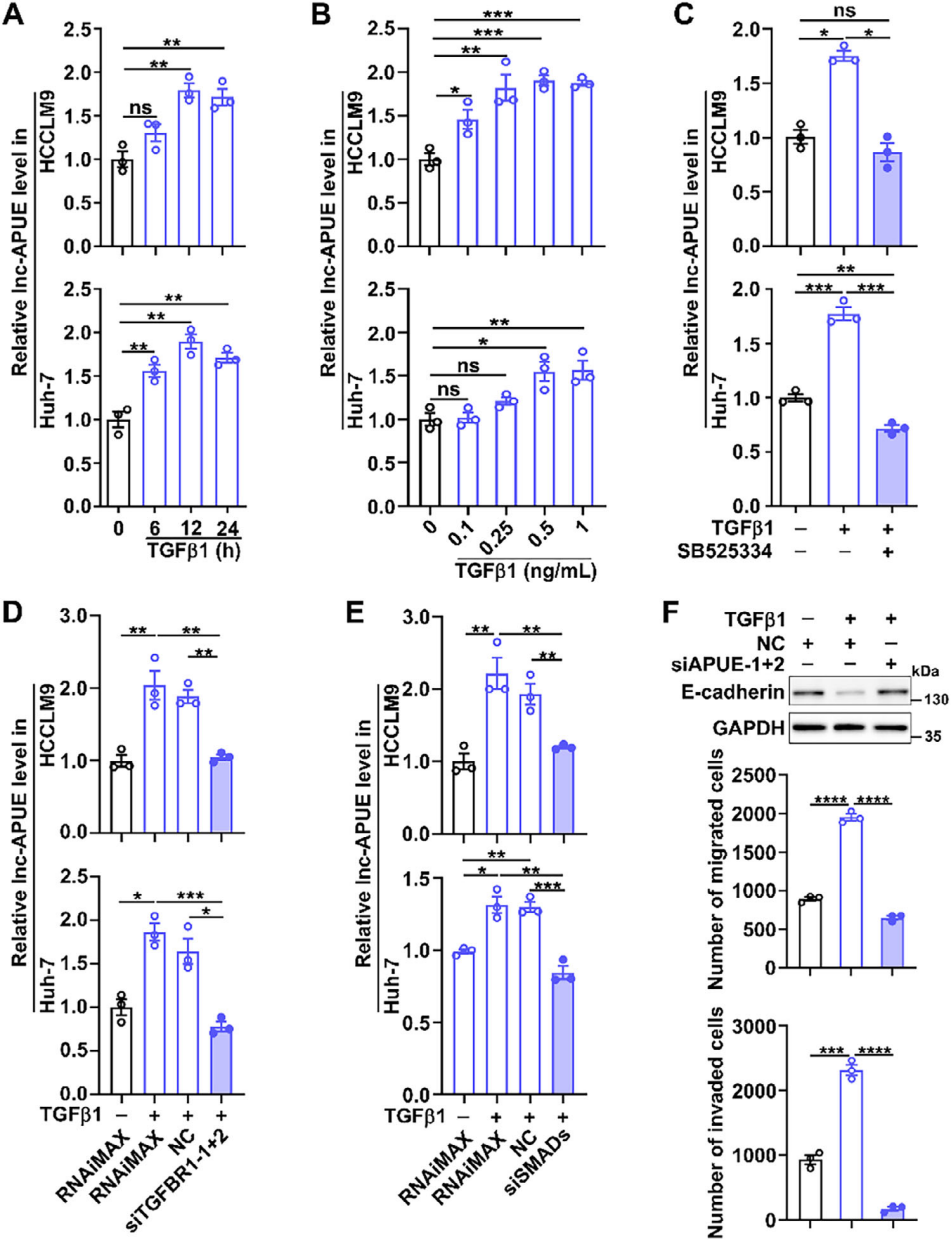
TGFβ1/SMAD signaling promotes tumor metastasis by enhancing lnc‐APUE expression. (A,B) TGFβ1 increased lnc‐APUE levels in hepatoma cells in a time‐ and dose‐dependent manner. HCCLM9 and Huh‐7 cells were untreated or treated with 1 ng/mL TGFβ1 for the indicated time (A) or with the indicated dose of TGFβ1 for 24 h (B). (C–E) Inhibition of TGFBR1 or simultaneous silencing of SMAD2/3/4 abolished the promotive role of TGFβ1 in lnc‐APUE expression. For (C), cells were untreated (‐) or treated with (+) TGFβ1 or TGFBR1 inhibitor (SB525334) for 12 h. For (D,E), HCCLM9 and Huh‐7 were transfected with the indicated RNA duplexes and then incubated without (‐) or with (+) TGFβ1 for 24 h before qPCR analysis. (F) Silencing lnc‐APUE abrogated the effects of TGFβ1 in reducing E‐cadherin levels and in promoting migration and invasion. HCCLM9 transfected with the indicated siRNAs were incubated without (‐) or with (+) TGFβ1 for 36 h, then subjected to western blotting (*Top* panel), migration (*middle* panel), and invasion (*bottom* panel) assays. Lnc‐APUE is abbreviated as “APUE”. RNAiMAX, cells exposed to Lipofectamine RNAiMAX without RNA. NC, negative control for siRNA. Data from at least three independent experiments are presented as mean ± SEM (A–F); *p* values were assessed by one‐way ANOVA (A–F). ^*^, *p* < 0.05; ^**^, *p* < 0.01; ^***^, *p* < 0.001; ^****^, *p* < 0.0001; ns, not significant.

Next, we examined whether TGFβ1/SMAD signaling regulated lnc‐APUE transcription. TGFβ1 treatment significantly increased phospho‐SMAD2 protein and upregulated both lnc‐APUE and CDH2 (Figure 6A,B; Figure S10C–E). Furthermore, the role of TGFβ1 in upregulating lnc‐APUE was blocked by either TGFBR1 inhibitor (SB525334) (Figure 6C), TGFBR1 knockdown (Figure 6D) or combined SMAD2/3/4 depletion (Figure 6E), implying that TGFβ1 may promote lnc‐APUE transcription through the canonical TGFBR1/SMAD2/3 pathway. Moreover, silencing lnc‐APUE disrupted the roles of TGFβ1 in repressing E‐cadherin expression and promoting tumor cell migration/invasion (Figure 6F; Figure S11), identifying lnc‐APUE as a critical mediator of TGFβ1‐driven tumor metastasis.

**FIGURE 7:**
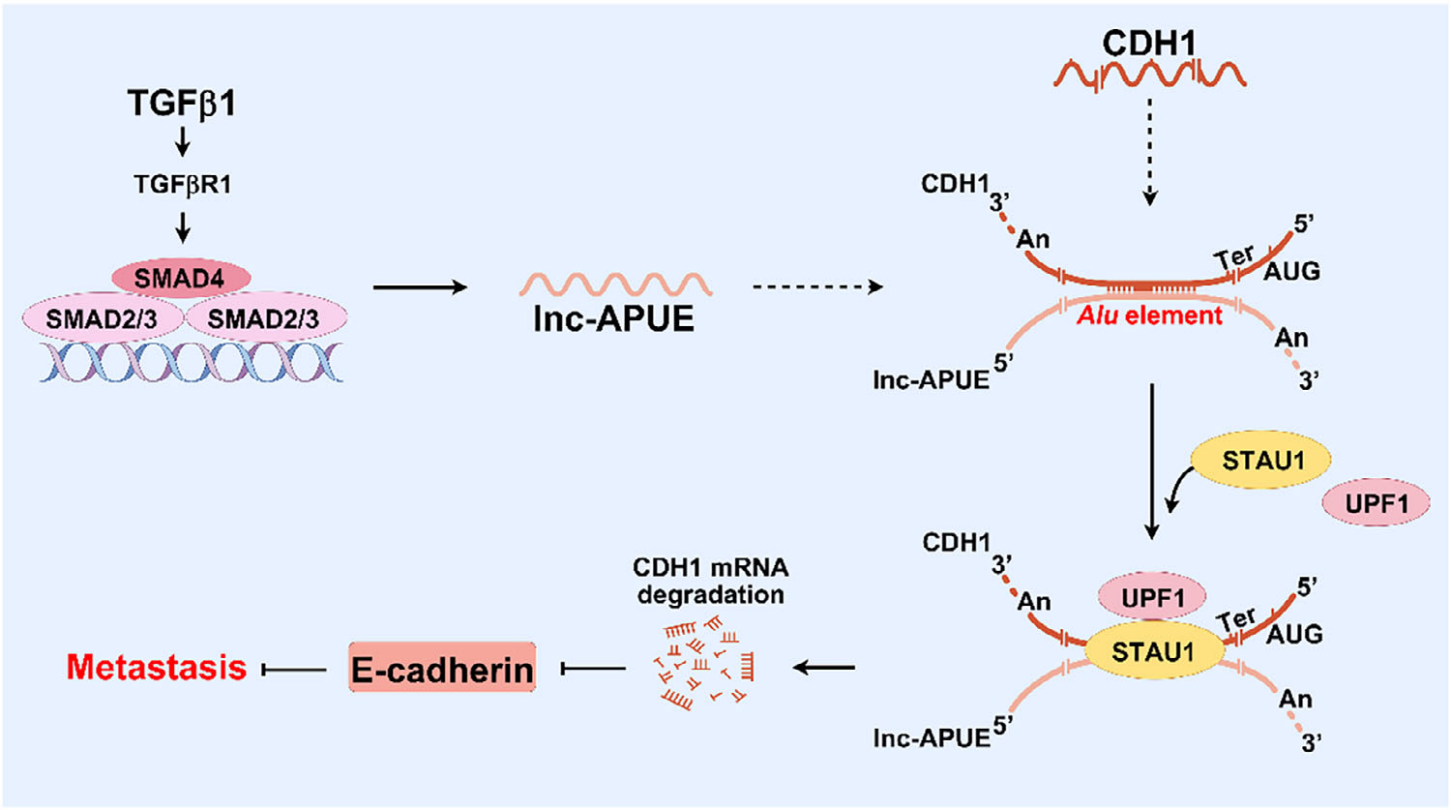
Schematic illustration of the TGFβ1/SMAD/lnc‐APUE/E‐cadherin axis and its regulatory role in tumor metastasis (By Figdraw).

Taken together, TGFβ1, an abundant cytokine in the tumor microenvironment, activates lnc‐APUE transcription via TGFBR1‐SMAD2/3 signaling. The upregulated lnc‐APUE then downregulates E‐cadherin by base‐pairing with the CDH1 3’UTR through the *Alu* element and triggering CDH1 mRNA decay via the SMD pathway, ultimately promoting tumor metastasis (Figure 7).

## 3 Discussion

*Alu* elements, the most prevalent transposable elements in primate genomes [5, 8, 28], primarily localize in introns, mRNA 3’UTRs, and lncRNAs [6, 8, 29]. These *Alu* RNAs have diverse biological functions, including miRNA sponging, alternative splicing of pre‐mRNA, and regulation of mRNA decay and translation [6, 8, 30]. Whether lncRNAs influence HCC progression via the *Alu* element remains unexplored.

Here, we demonstrated that lnc‐APUE induced CDH1 mRNA decay through the *Alu* element and thereby promoted HCC metastasis, based on the following evidence: (1) Bioinformatics analysis, RNA‐RNA EMSA assay, luciferase reporter assays, S1m‐tagged RNA affinity purification, RIP assay, and biochemical analyses validated the interaction between the *Alu* element of lnc‐APUE and CDH1 3’UTR, and its effect in reducing CDH1 mRNA stability. (2) Both gain‐ and loss‐of‐function studies showed that lnc‐APUE enhanced hepatoma cell metastasis in vitro and in vivo, and silencing CDH1 abrogated the effect of lnc‐APUE knockdown, suggesting a role of the lnc‐APUE/CDH1 regulatory axis in metastasis. (3) Deletion or mutation of the *Alu* element within lnc‐APUE abrogated the functions of lnc‐APUE in promoting CDH1 mRNA decay and metastasis, further confirming that the *Alu* element and the specific interaction of lnc‐APUE and CDH1 are essential for lnc‐APUE to exert metastasis‐promoting function.

It is well accepted that multiple signaling pathways frequently converge to regulate identical cellular processes, enabling precise control mechanisms. We also acknowledge that lnc‐APUE may interact with additional molecular partners through distinct mechanisms. In our previous study, we showed that lnc‐APUE enhanced cell proliferation by relieving the repression of miR‐20b on E2F1 [26]. Beyond its well‐known role in driving the cell cycle, E2F1 also amplifies metastatic potential. E2F1 enhances TGFβ1 protein levels via trans‐activating troponin C‐1 (TNNC1) expression [31], and promotes the transcription of Snail1, a target of the TGFβ1/SMAD pathway, via recruiting histone demethylases to the Snail1 promoter [32]. Notably, the pro‐metastatic capacity of lnc‐APUE was attenuated by mutating the miR‐20b‐binding sites in lnc‐APUE (APUE‐20b‐mut) (Figure S12A) or by knocking down E2F1 (Figure S12B), confirming that lnc‐APUE may also promote metastasis via regulating the miR‐20b/E2F1 axis. We therefore propose that silencing lnc‐APUE may suppress tumor cell migration and invasion by directly increasing CDH1 mRNA and decreasing E2F1 protein levels. These synergistic effects likely underlie the pronounced efficacy of siAPUE in countering the roles of TGFβ1 treatment observed in this study. Interestingly, one of the two miR‐20b seed sites (located at 301–324 nt) falls within the 290–402‐nt region of lnc‐APUE that is responsible for its interaction with CDH1 (Figure S1B). Future studies should explore whether miR‐20b binding to lnc‐APUE affects the lnc‐APUE–CDH1 mRNA interaction, and conversely, whether CDH1 mRNA binding to lnc‐APUE influences the lnc‐APUE–miR‐20b interaction.

It is widely demonstrated that many of lncRNAs play important regulatory roles in gene expression and cellular activity, although they are generally expressed at low levels [13, 33]. Emerging evidence suggests that lncRNAs may regulate target molecules through a catalytic‐like recycling mechanisms rather than a static one‐to‐one binding, explaining how minimal cellular copy numbers enable their function [12, 33, 34]. It has been shown that a 1:70 lncRNA:protein ratio is sufficient for lncRNA's functional regulation, with effects persisting even at 1:1000 ratio despite delayed kinetics [34]. Consistently, we found that knocking down STAU1 or UPF1 increased CDH1 mRNA levels without affecting lnc‐APUE levels (Figure S13). Therefore, we propose that lnc‐APUE may be recycled to induce CDH1 mRNA decay.

E‐cadherin, encoded by CDH1, is essential for maintaining epithelial cell‐cell adhesion and its loss facilitates cancer cells to detach from primary site, and to migrate, invade and metastasize [27, 35, 36]. Downregulation of E‐cadherin is frequently observed in different cancer types and is associated with poorer prognosis [37, 38]. The mechanisms responsible for E‐cadherin reduction include loss of genomic sequence [39], hypermethylation of the CDH1 promoter [39], and overexpression of transcriptional repressors (e.g. Snail, Slug and Twist) [40] or splicing factor (UPF3B‐S) [41] or gene‐silencing microRNA (miR‐9) [38, 39, 42]. However, the role of *Alu* element in regulating CDH1 mRNA stability is unknown. Here, we identified an *Alu* element in lnc‐APUE that base‐paired with the *Alu* element within the CDH1 3’UTR to destabilize CDH1 mRNA through the SMD pathway. These findings indicate that the *Alu* element‐mediated mRNA decay may represent a novel mechanism underlying E‐cadherin downregulation in tumors.

TGFβ1, a pleiotropic cytokine, plays a pivotal role in promoting cancer metastasis [43, 44]. Canonical TGFβ1 signaling is initiated when TGFβ1 binds to the type 2 TGFβ receptor (TGFBR2), thereby recruits and phosphorylates TGFBR1, resulting in phosphorylation and activation of SMAD2/3, which bind with SMAD4 and translocate to the nucleus to regulate gene transcription [45, 46]. TGFβ1 is abundant in HCC microenvironment and correlated with poor prognosis of patients [47]. In previous study, we identified the core promoter of lnc‐APUE and showed that the −212–−140‐bp region of the lnc‐APUE promoter contained the HNF4α responsive element, and decrease of HNF4α in HCC cells led to upregulation of lnc‐APUE transcription. In this study, we found that TGFβ1 treatment upregulated lnc‐APUE transcription through the canonical TGFBR1/SMAD2/3 pathway, by which SMAD2 interacted with the lnc‐APUE promoter, suggesting that deregulation of different transcription factors jointly increases lnc‐APUE transcription during HCC development. It has been shown that TGFβ1 also downregulates HNF4α expression [48, 49], indicating that TGFβ1 may increase lnc‐APUE expression directly through SMAD2/3 and indirectly through HNF4α in cancer cells.

The TGFβ1/SMAD signaling has been disclosed to repress CDH1 transcription by upregulating transcription factors ZEB1, Snail, and Slug [50]. Emerging evidence indicates that SMAD‐driven induction of lncRNAs and circRNAs adds another layer of repression. For instance, some circRNAs (e.g., hcirc_0057481) function as competing endogenous RNAs to alleviate miRNA‐mediated inhibition of ZEB1/2 [51]. LncRNA SNHG8, when depleted, destabilizes CDH1 mRNA [52]. The present study further reveals that the TGFβ1/SMAD pathway may downregulate CDH1 at the post‐transcriptional level through lnc‐APUE‐mediated mRNA decay, thereby enriching our understanding of the mechanism by which TGFβ1 governs CDH1 expression.

In summary, we identify a TGFβ1/SMAD/lnc‐APUE/E‐cadherin regulatory axis and elucidate its role in promoting HCC metastasis, which may be exploited for HCC treatment.

## 4 Experimental Section

Additional information is provided in the Supporting Information.

### 4.1 Reagents

Reagents were purchased as follows: TGFβ1 (240‐B‐002, R&D Systems, Minneapolis, MN, USA); SB525334 (S1476, Selleckchem, Houston, TX, USA); actinomycin‐D (ActD, 15021 S, Cell Signaling Technology, CST, Beverly, MA, USA). Unless otherwise indicated, the final concentrations used were 1 ng/mL TGFβ1, 2 µm SB525334, or 5 µg/mL ActD.

### 4.2 Human Tissues

Human HCC and adjacent non‐tumor liver tissues were obtained from patients who underwent curative resection at Sun Yat‐sen University Cancer Center. The selection criteria included: (1) HCC diagnosis with pathological confirmation; (2) Accessibility of matched fresh‐frozen tissues pairs; (3) No prior anticancer treatment before resection. Written informed consent was obtained from each patient, and the study protocol was approved by the Ethics Committee of Sun Yat‐sen University Cancer Center (GZR2019‐086). All specimens were snap‐frozen in liquid nitrogen immediately after collection. Baseline clinical characteristics of the study cohort are summarized in Table S1.

### 4.3 RNA Oligoribonucleotides and Plasmid Construction

Small interfering RNA (siRNA) targeting human gene *lnc‐APUE* (NR_105045.1), *CDH1* (NM_004360.5), *STAU1* (NM_017453.4), *UPF1* (NM_001297549.2), *TGFBR1* (NM_004612.4), *SMAD2* (NM_005901.6), *SMAD3* (NM_005902.4), *SMAD4* (NM_005359.6), and *E2F1* (NM_005225) were denoted as siAPUE, siCDH1, siSTAU1, siUPF1, siTGFBR1, siSMAD2, siSMAD3, siSMAD4 and siE2F1, respectively. All siRNAs were purchased from RIBOBIO (Guangzhou, China). The negative control for siRNAs was designed to have no homology to any human genome sequence. Sequences of all duplexes are listed in Table S2.

Lentivirus expression vectors, including pCDH‐APUE, pCDH‐APUE‐∆*Alu*, pCDH‐APUE‐mut, pCDH‐STAU1‐Flag, pCDH‐S1m‐APUE, pCDH‐S1m‐APUE‐∆*Alu*, pCDH‐shNC, pCDH‐shAPUE were generated based on pCDH‐CMV‐MCS‐EF1‐copGFP (System Biosciences, Palo Alto, CA, USA; RRID: Addgene_99729), which expresses copGFP and was designated as pCDH‐Ctrl in this study.

Luciferase reporter plasmid pSBE, which contains twelve tandem SBEs, was generously provided by Peter ten Dijke (Leiden University Medical Center, Leiden, the Netherlands). The luciferase reporter vectors psi‐CDH1‐3’UTR and psi‐CDH1‐3’UTR‐∆*Alu* were generated using the dual‐luciferase reporter plasmid psiCHECK2 (Promega, Madison, WI, USA; RRID: Addgene_110401) as a backbone. In this system, *Renilla* luciferase serves as the experimental reporter, while *Firefly* luciferase provides the internal control. *Firefly* luciferase reporter vectors containing fragments of the putative lnc‐APUE promoter were constructed using the pGL3‐basic vector (Promega). These vectors included P(‐1553/+70), P(‐889/+70), P(‐505/+70), P(‐212/+70), P(delSBE1/2), P(delSBE3) and P(mutSBE1/2). For normalization, the pRL‐TK vector (E2241, Promega) expressing *Renilla* luciferase was used as a control.

### 4.4 Cell Lines

Human embryonic kidney cell line HEK293T (ATCC, CRL‐3216; RRID: CVCL_0063), human hepatoma cell lines including HCCLM9 (RRID: CVCL_A5CU) [53, 54], Huh‐7 (RRID: CVCL_0336) [55], SK‐Hep‐1 (ATCC, HTB‐52; RRID: CVCL_0525), and SNU‐449 (ATCC, CRL‐2234; RRID: CVCL_0454) were used. SNU‐−449 and SK‐Hep‐1 cells were maintained in RPMI 1640 medium (Corning, New York, USA) containing 10% fetal bovine serum (FBS; Hyclone, Logan, UT), while the others were grown in Dulbecco's modified Eagle's medium (DMEM; Life Technologies, Gaithersburg, MD, USA) supplemented with 10% FBS.

Stable cell lines, which were generated in HCCLM9, Huh‐7, SNU‐449 and SK‐Hep‐1 cell lines via lentivirus infection, included sublines stably expressing STAU1‐Flag or wild‐type lnc‐APUE sequence (LM9‐STAU1‐Flag, LM9‐APUE, Huh‐APUE, SNU‐APUE, SK‐APUE), lnc‐APUE with *Alu* element deletion (LM9‐APUE‐∆*Alu*, SNU‐APUE‐∆*Alu*), lnc‐APUE with *Alu* element complementary mutation (SNU‐APUE‐mut) or lnc‐APUE with miR‐20b‐binding site mutation (SK‐APUE‐20b‐mut) [26], sublines with stable expression of S1m‐tagged wild‐type lnc‐APUE (LM9‐S1m‐APUE) or S1m‐tagged lnc‐APUE with *Alu* element deletion (LM9‐S1m‐APUE‐∆*Alu*), sublines with stable silencing of lnc‐APUE (LM9‐shAPUE) or their control lines (LM9‐shNC).

### 4.5 Cell Transfection

Twenty‐five nm of RNA duplex were transfected using Lipofectamine RNAiMAX (Invitrogen, Carlsbad, CA, USA). Transfection of plasmids was conducted by Lipofectamine 2000 (Invitrogen).

### 4.6 Lentivirus Production and Infection

HEK293T cells were transfected with the lentiviral expression vector containing the target sequence along with packaging plasmids. The lentiviral supernatant was collected 48 h later. Human hepatoma cell lines were infected with lentiviral supernatant supplemented with 10 µg/mL polybrene (Millipore, Billerica, MA, USA) for 72 h.

### 4.7 Analysis of Gene Expression

The levels of target genes were analyzed by real‐time quantitative polymerase chain reaction (qPCR), northern blotting, western blotting, or immunohistochemical staining. The primers used for qPCR and probes for northern blotting are listed in Table S2.

### 4.8 Luciferase Reporter Assay

Cells were seeded in a 48‐well plate at a density of 3×10^4^ and incubated for 24 h before transfection. Luciferase activity was measured 48 h post‐transfection using a dual luciferase reporter assay system (Promega).

To examine the binding capacity of lnc‐APUE and STAU1 to CDH1 3’UTR, cells were co‐transfected with 25 nm RNA duplex and 25 ng luciferase reporter vectors (psi‐CDH1‐3’UTR or psi‐CDH1‐3’UTR‐∆*Alu*). To test the role of STAU1 or UPF1 in lnc‐APUE‐mediated repression of CDH1‐3’UTR reporter activity, the stable cell lines SNU‐APUE/Ctrl were co‐transfected with 25 nm RNA duplex and 25 ng psi‐CDH1‐3’UTR vector. *Renilla* luciferase activity was normalized to the corresponding *Firefly* luciferase activity.

To examine whether the TGFβ1 signaling pathway is functional and inducible in our experimental system, cells were co‐transfected with 200 ng pSBE and 50 ng pRL‐TK for 24 h, followed by incubation with 1 ng/mL TGFβ1 for 12 h before the luciferase activity assay. To characterize the lnc‐APUE promoter, cells were co‐transfected with 50 ng of *Firefly* luciferase reporter vector and 25 ng of pRL‐TK, followed by incubation without or with 1 ng/mL of TGFβ1 for another 24 h before the luciferase activity assay. The *Firefly* luciferase activity of each sample was normalized to the *Renilla* luciferase activity.

### 4.9 RNA‐RNA EMSA

Custom‐synthesized *Alu* RNA for lnc‐APUE and CDH1 3’UTR (Tsingke Biotechnology Co., Ltd., Beijing, China) was used as the probes in EMSA adapted from an established protocol [56]. Briefly, for the binding reaction, 10 µL mixtures containing biotin‐labeled CDH1 3’UTR‐*Alu* RNA, recombinant RNasin (Promega, N2515), wild‐type or mutant lnc‐APUE‐*Alu* RNA, and 1X structure buffer (Invitrogen RNase T1 enzyme kit, AM2283) were incubated at 37°C for 1 h and cooled on ice for 10 min. Five microliters of 50% glycerol (v/v) was added to each reaction to increase sample density and facilitate gel loading. The samples were then separated on a native 5% (w/v) polyacrylamide gel that was pre‐run at 100 V for 30 min at 4°C in 0.5X TBE buffer. The electrophoresis was performed at 100 V for 1 h at 4°C in the same buffer. Nucleic acids were visualized by staining the gel with 3× GS‐GelRed (Genesand Biotech, GL802) for 30 min and imaging with a gel documentation system. Probe sequences are listed in Table S2.

### 4.10 S1m‐Tagged RNA Affinity Purification

S1m‐tagged RNA affinity purification assay, as described previously [26], was employed to examine the interaction between lnc‐APUE and the CDH1 3’UTR. HCCLM9 (2×10^7^) sublines with stable expression of APUE, S1m‐APUE or S1m‐APUE‐∆*Alu* were washed with pre‐chilled 1X PBS three times, collected by centrifugation at 500× g for five min, dissolved in 8 mL of 1× PBS containing 0.37% formaldehyde and rotated at room temperature (RT) for 5 min to crosslink cellular components, followed by treatment with 250 mm glycine at RT for another 5 min to abort crosslinking reaction. Cell pellets were washed two times with 5 mL ice‐cold 1X PBS, resuspended in ice‐cold lysis buffer (150 mm NaCl, 10 mm HEPES at pH 7.4, 3 mm MgCl_2_, 10% glycerol, 1% NP‐40, 2 mm DTT plus RNase and proteinase inhibitors), followed by incubation on ice for 10 min, and then sonicated six times with a Bioruptor. The supernatant was collected by centrifugation (18 000×*g*, 4°C, 10 min) and pre‐cleared by incubation with 40 µL avidin agarose beads (20219, Pierce, Rockford, IL, USA) at 4°C for 1 h to remove the background; then the pre‐cleaned cell lysate was transferred to a new tube and 1/20 of the lysate served as the RNA input. The remaining lysate was treated with 30 µL streptavidin Dynabeads (Invitrogen) and rotated at 4°C for 3 h to pulldown S1m‐tagged RNA‐RNA complexes. The beads were collected, washed five times with 500 µL ice‐cold RNase‐free washing buffer (50 mm HEPES at pH 7.4, 400 mm NaCl, 0.1% TritonX‐100, 10% glycerol, 2% NP‐40, 1 mm EDTA, 1 mm PMSF, and 1 mm DTT) for 5 min each at 4°C with rotation. Next, 100 µL RNase‐free elution buffer (50 mm HEPES at pH 7.4, 5 mm EDTA, 100 mm NaCl, 1% SDS and 10 mm DTT) was added, and the mixture was heated at 70°C for 45 min to reverse formaldehyde crosslinking. RNAs were extracted from the precipitates by TRIzol reagent (Invitrogen) and detected by qPCR.

### 4.11 RIP Assay

Following transfection with or without RNA duplex, LM9‐STAU1‐Flag cells were washed three times with 5 mL pre‐chilled 1X PBS, and then incubated with 0.5% formaldehyde at RT for 10 min to crosslink cellular components; the reaction was quenched with 250 mm glycine at RT for 5 min. Cells were washed three times with 5 mL ice‐cold 1X PBS, lysed with RIP lysis buffer (25 mm Tris‐HCl at pH 7.4, 150 mm NaCl, 1 mm EDTA, 1% NP‐40, 5% glycerol) supplemented with protease and RNase inhibitors, and incubated at 4°C for 10 min. The samples were sonicated four times with a Bioruptor (Diogenode, Liege, Belgium), followed by centrifugation at 13 000 g, 4°C for 10 min. The supernatants were pre‐cleared by 20 µL of Dynabeads Protein G (10004D, Invitrogen), incubated with 4 µg of antibody at 4°C for 4 h, followed by addition of 40 µL Dynabeads G (Invitrogen) and further incubation at 4°C for another 2 h. The beads were collected and washed five times with a high‐salt RIP lysis buffer (25 mm Tris‐HCl at pH 7.4, 500 mm NaCl, 1 mm EDTA, 1% NP‐40, and 5% glycerol) and then resuspended in 50 µL of 100 mm glycine (pH 3.0) to release the immunoprecipitated complex. Then, the immunoprecipitates were treated with 5 µL of 1 m Tris‐HCl (pH 8.0) and 5 µg proteinase K, and incubated sequentially at 55°C for 1 h and then at 70°C for 45 min to reverse the formaldehyde crosslinking. Total RNA was extracted from the eluates, followed by qPCR analysis for target genes. The antibodies used for the RIP assay included rabbit mAb against Flag (F1804, Sigma–Aldrich; RRID: AB_262044) and rabbit isotype‐matched control IgG (A7016, Beyotime, Shanghai, China; RRID: AB_2905533).

### 4.12 Migration and Invasion Assays

The in vitro migration and invasion capacities of tumor cells were evaluated using Transwell chambers with an 8‐µm pore size polycarbonate membrane (Corning). For invasion assays, the upper chamber was pre‐coated with Matrigel (3432‐005‐01, R&D Systems) to establish matrix barriers on the membrane. Tumor cells were resuspended in 100 µL of serum‐free medium and seeded into the upper chamber with or without Matrigel‐coating, while 600 µL of 10% FBS‐containing medium was added to the lower chamber. After incubation at 37°C for 10 h, cells were fixed with 4% paraformaldehyde and stained with 0.1% crystal violet. The cells remaining on the upper surface of the membrane were removed, and all cells that migrated or invaded to the lower surface of the membrane were counted in five randomized fields under a light microscope.

### 4.13 Mouse Xenograft Models

Male NOD‐Prkdc^em26Cd52^Il2rg^em26Cd22^/Nju (NCG) mice and male BALB/c nude mice at 4–5 weeks of age were used. All experimental procedures were approved by the Institutional Animal Care and Use Committee of Sun Yat‐sen University (SYSU‐IACUC‐2021‐B0479; SYSU‐IACUC‐2021‐B1317). HCCLM9‐derived stable cell lines (shNC, shAPUE, Ctrl, APUE, or APUE‐∆*Alu*) (4.0×10^5^) were resuspended in 25 µL of serum‐free DMEM/Matrigel (1:1) and inoculated under the capsule of the left hepatic lobe of mice. Four weeks later, mice were humanely euthanized, and the primary tumor, livers, and lungs were collected. The volume of tumor was calculated with formula = length×width^2^/2.

### 4.14 ChIP Assay

HCCLM9 cells were treated with 0.5 ng/mL of TGFβ1 for 2 h, cross‐linked with 0.75% formaldehyde for 10 min at RT. Chromatin was sheared by sonication to generate ∼500‐bp fragments, followed by immunoprecipitation with anti‐SMAD2 antibody (5339, CST; RRID: AB_10626777). The immunoprecipitated DNAs were recovered, purified, and amplified by semi‐quantitative PCR or qPCR with primers listed in Table S2.

### 4.15 Bioinformatic and Statistical Analysis

The mRNA expression profiling of paired HCC and noncancerous liver tissues was obtained from Gene Expression Omnibus (GEO; RRID: SCR_005012) datasets (accession number GSE77314 and GSE115018). The mRNA profile of STAU1‐immunoprecipitates was obtained from GSE8438. The software Repeatmasker (http://www.repeatmasker.org/cgi‐bin/WEBRepeatMasker; RRID: SCR_012954) was used to identify the *Alu* element within the lnc‐APUE sequence. The IntaRNA (http://www.rnainter.org/IntaRNA/; RRID: SCR_027258) and NCBI blastn algorithm (https://www.blast.ncbi.nlm.nih.gov/Blast.cgi; RRID: SCR_001598) was used to predict the potential base‐pairing between the lnc‐APUE *Alu* element with *Alu* elements in the 3’UTRs of candidate mRNAs.

All analyses were conducted using GraphPad Prism version 8.0 (GraphPad Software, Inc., San Diego, CA, USA; RRID: SCR_002798). The data from at least three independent assays were presented as the mean ± standard error of the mean (SEM). The differences between two groups were analyzed using Student's *t*‐test. For comparison among more than two groups, one‐way analysis of variance (ANOVA) was used to evaluate the impact of one independent variable, while two‐way ANOVA was applied to assess two independent variables. All statistical tests were two‐sided, and *p* < 0.05 was defined as significant.

## Author Contributions

S.Y.L. designed and performed experiments, discussed and interpreted the data, and wrote the manuscript. J.H.H., Y.H.L., J.Z.H., T.T.W., Y.L.C., M.Z.W., and W.W. performed experiments and interpreted the data. S.M.Z., Y.Z., and J.E.Y. supervised and designed the study, discussed and interpreted the data, and wrote the manuscript. All authors read and approved the final manuscript.

## Conflicts of Interest

The authors declare no conflicts of interest.

## Supporting information




**Supporting File**: advs74161‐sup‐0001‐SuppMat.docx.

## Data Availability

Data sharing is not applicable to this article as no new data were created or analyzed in this study.
