# Supplementary material for: TGFβ1 Activates Lnc‐APUE to Promote Tumor Metastasis via the Alu Element‐Driven STAU1‐Mediated Decay of CDH1 mRNA
Source: Adv Sci (Weinh). 2026 Feb 3;13(20):e18731. doi: 10.1002/advs.202518731 (PMC13067822; doi:10.1002/advs.202518731)
Supplement: Supplementary file 1 — Supporting File: advs74161‐sup‐0001‐SuppMat.docx. [file ADVS-13-e18731-s001.docx]

**Supplementary Information**

TGFβ1 Activates Lnc-APUE to Promote Tumor Metastasis via *Alu* Element-driven STAU1-mediated Decay of CDH1 mRNA

*Song-Yang Li^#^, Jia-Hui Huang^#^, Jin-E Yang, Yi-Hang Li, Juan-Zhen Hong, Ting-Ting Wang, Ying-Lei Chi, Meng-Zhi Wu, Wei Wang, Ying Zhu*, Shi-Mei Zhuang**

**Inventory of supplementary data**

1. Supplementary Experimental Section...………….…..Page 2 - 6

2. Supplementary Figures and Legends...………….…......Page 7 - 25

3. Supplementary Tables...………….…..………….….....Page 26 - 31

**Supplementary Experimental Section**

**Plasmid construction**

The following plasmids were used: lentivirus expression vectors, including pCDH-APUE, pCDH-APUE-∆*Alu*, pCDH-APUE-mut, pCDH-S1m-APUE, pCDH-S1m-APUE-∆*Alu*, pCDH-shNC, pCDH-shAPUE, pCDH-STAU1-Flag; luciferase reporters, including psi-CDH1-3’UTR, psi-CDH1-3’UTR-∆*Alu*, P(-1553/+70), P(-889/+70), P(-505/+70), P(-212/+70), P(delSBE1/2), P(delSBE3), P(delSBE1/2/3), P(mutSBE1/2).

To produce pCDH-APUE or pCDH-APUE-∆*Alu*, full-length lnc-APUE (1123-nt) with or without the *Alu* element (position in lnc-APUE: 290-402 nt) was inserted into the *Eco*RI/*Swa*I sites of the lentivirus expression vector pCDH-CMV-MCS-EF1-copGFP (System Biosciences, Palo Alto, CA, USA), which contained a copGFP expression cassette and was designated as pCDH-Ctrl in this study. The plasmid pCDH-APUE-mut bearing complementary nucleotide substitutions within the *Alu* sequence was constructed by GentleGen (Beijing, China). To create pCDH-STAU1-Flag, the coding sequence of STAU1 fused with a Flag-tag at its C-terminus was inserted into the *Xba*I/*Not*I sites of pCDH-Ctrl vector.

To accomplish S1m-tagged RNA affinity purification assay, pCDH-S1m was constructed by inserting four tandem S1m sequences, which encoded the streptavidin-binding RNA aptamer, into the *Xba*I/*EcoR*I sites in pCDH-Ctrl^1^. Full-length lnc-APUE with or without the *Alu* element (290-402 nt) was then inserted into the *EcoR*I/*Swa*I sites downstream of the 4×S1m sequence in pCDH-S1m to create pCDH-S1m-APUE or pCDH-S1m-APUE-∆*Alu*.

To create pCDH-shNC and pCDH-shAPUE plasmids, the complementary oligonucleotides that contained both sense and antisense siRNA sequences (shNC: 5’-TGAATTAGATGGCGATGTT-3’; shAPUE: 5’-GGACTTGAGTGAGATGTCA-3’), the spacer sequence (5’-CTCGAG) and the flanking *EcoR*I and *BamH*I sites were chemically synthesized, annealed and then inserted into the *EcoR*I/*BamH*I sites in the pCDH-U6 plasmid, which was produced by replacing the CMV promoter of the pCDH-CMV-MCS-EF1-copGFP plasmid with the U6 promoter.

To verify whether lnc-APUE down‐regulated CDH1 by binding to the *Alu* element (position in CDH1 3’UTR: 857-1003 nt) in the CDH1 3’UTR, psi-CDH1-3’UT­R and psi-CDH1-3’UTR-∆*Alu* were generated. To generate psi-CDH1-3’UTR construct, a 1000-bp fragment encompassing the *Alu* sequence within the CDH1 3’UTR along with a 400-bp flanking sequences upstream and downstream of the *Alu* element, was amplified and inserted into the *Xho*I/*Not*I sites downstream of the *Renilla* luciferase stop codon in the psiCHECK2 dual-luciferase reporter vector (Promega, Madison, WI, USA). This construct also expresses *Firefly* luciferase as an internal control reporter to normalize transfection efficiency. The psi-CDH1-3’UTR-∆*Alu* construct was generated by deleting the *Alu* sequence from the psi-CDH1-3’UTR vector using PCR.

To investigate the regulation of lnc-APUE transcription, we generated the promoter vector pGL3-P(-1553/+70) by cloning the -1553 to +70-bp sequence of the lnc-APUE promoter into the *Kpn*I/*Hind*III sites upstream of the *Firefly* luciferase gene in a pGL3-basic vector (Promega). Sequential 5’-deletion constructs of the lnc-APUE promoter were created via fusion PCR using pGL3-P(-1553/+70) as the template. Additionally, the luciferase reporter vectors P(delSBE) and P(mutSBE), carrying deletion or mutation of the predicted SMAD-binding element (SBE) in the lnc-APUE promoter, respectively, were constructed by fusion PCR using pGL3-P(-505/+70) as the template.

All plasmids were verified by direct DNA sequencing. The sequences of primers are listed in Supplementary Table S2.

***Analysis of gene expression***

Real-time quantitative polymerase chain reaction (qPCR) and northern blotting were performed to detect RNA levels. Total RNA was extracted by TRIzol reagent (Invitrogen), and reverse-transcribed using M-MLV reverse transcriptase (M1701, Promega). qPCR was conducted on a LightCycler 480 (Roche Diagnostics, Germany) using 2×SYBR Green qPCR Master Mix (B21202, Bimake, Houston, TX, USA). All reactions were performed in duplicate. The cycle threshold (Ct) values differed by less than 0.3 between duplicate wells. The level of target gene was normalized to that of endogenous reference gene GAPDH, which yielded a 2^-ΔΔCt^ value.

For northern blotting, 15 μg (Huh-7) or 13 μg (SNU-449) of total RNA was denatured at 65 °C for 15 min and separated on 1% agarose gels containing 18% formaldehyde and 1× MOPS buffer (R0221, Beyotime). After electrophoresis, RNA was transferred to nylon membranes (RPN303B, GE Healthcare), UV-cross-linked, and hybridized with biotin-labeled probes in hybridization solution (R0229, Beyotime) at 42 °C for 16 h. The membranes were washed three times in 2× SSC/0.1% SDS at 42 °C for 5 min each, followed by two washes with 0.1×SSC/0.1% SDS at 65 °C for 5 min each. After blocking with blocking buffer (D3308B, Beyotime) for 30 min at room temperature (RT), the membranes were incubated with anti-biotin-HRP (1:2000, A0305, Beyotime) for 30 min at RT. Excess antibody was removed by brief washing in washing buffer (100 mM Tris-HCl, 150 mM NaCl, and 0.3% Tween-20). Signals were developed using an ECL kit (1705061, Bio-Rad). The sequences of qPCR primers and northern blotting probes are listed in Supplementary Table S2.

Western blotting was conducted to examine the protein levels. The antibodies used in this study included rabbit monoclonal antibody (mAb) against E-cadherin (3195, Cell Signaling Technology, CST, Beverly, MA, USA; RRID: AB_2291471), Phospho-SMAD2 (3108, CST; RRID: AB_490941), SMAD2 (5339, CST; RRID: AB_10626777), N-cadherin (BA0673, Boster; RRID: AB_3717558); mouse polyclonal antibody against GAPDH (BM1623, Boster; RRID: AB_2885058).

For immunohistochemical staining, sections of formalin-fixed, paraffin-embedded tissues were cut into a 3.5 μm section and mounted on polylysine-coated slides. The slides underwent dewaxing, endogenous peroxidase activity quenching with 0.3% hydrogen peroxide, and antigen retrieval through high-pressure heating in sodium citrate buffer (pH 6.0) for 10 min, followed by overnight incubation at 4 ℃ with primary antibodies. A rabbit mAb against E-cadherin (3195, CST) was applied at a dilution of 1:200. The immunostaining procedure was carried out using the ChemMate DAKO EnVision Detection Kit, Peroxidase/DAB, Rabbit/Mouse (K5007, Agilent Technologies, Inc., CA, USA), yielding a brown precipitate at the antigen sites. The sections were then counterstained with hematoxylin (DH0005, Leagene, Beijing, China) and coverslipped with a non-aqueous mounting medium. Each assay included a negative control without primary antibody. The stained sections were scanned using a digital scanner (Aperio VERSA 200, Leica, Germany), and the relative area of E-cadherin staining to the total tissue area was assessed using Aperio software (Leica).

1. Li SY, Zhu Y, Li RN, Huang JH, You K, Yuan YF, et al. LncRNA Lnc-APUE is Repressed by HNF4α and Promotes G1/S Phase Transition and Tumor Growth by Regulating MiR-20b/E2F1 Axis*.* *Adv Sci (Weinh)* **2021**;8:2003094.

**Supplementary Figures and Legends**


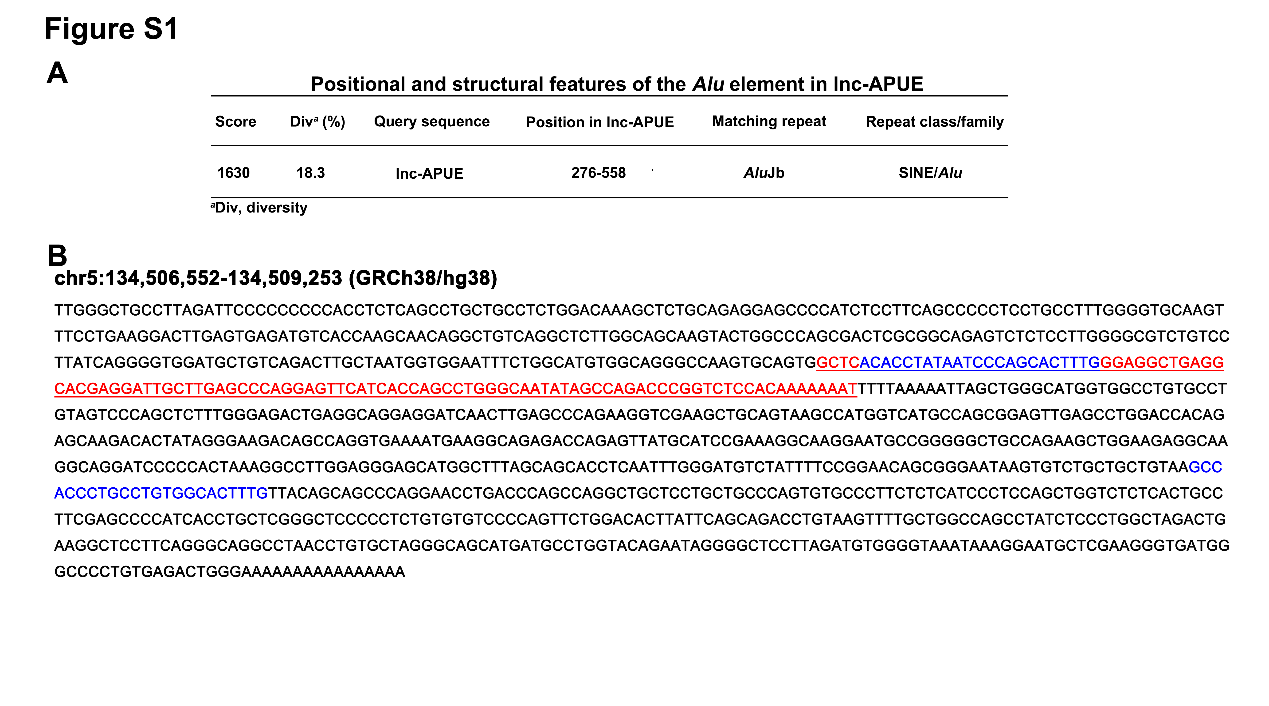


Supplementary Fig. S1. The *Alu* element in lnc-APUE. (A) Bioinformatics analyses using software Repeatmasker predicted a single *Alu* element in lnc-APUE. Div (diversity) quantified the differences between the *Alu* sequence of lnc-APUE and the canonical *Alu*Jb consensus sequence. (B) The full-length lnc-APUE. The *Alu* element (290~402 nt) is highlighted in red and underlined, and the miR-20b binding sites (301~324 nt and 771~790 nt) is in blue font.


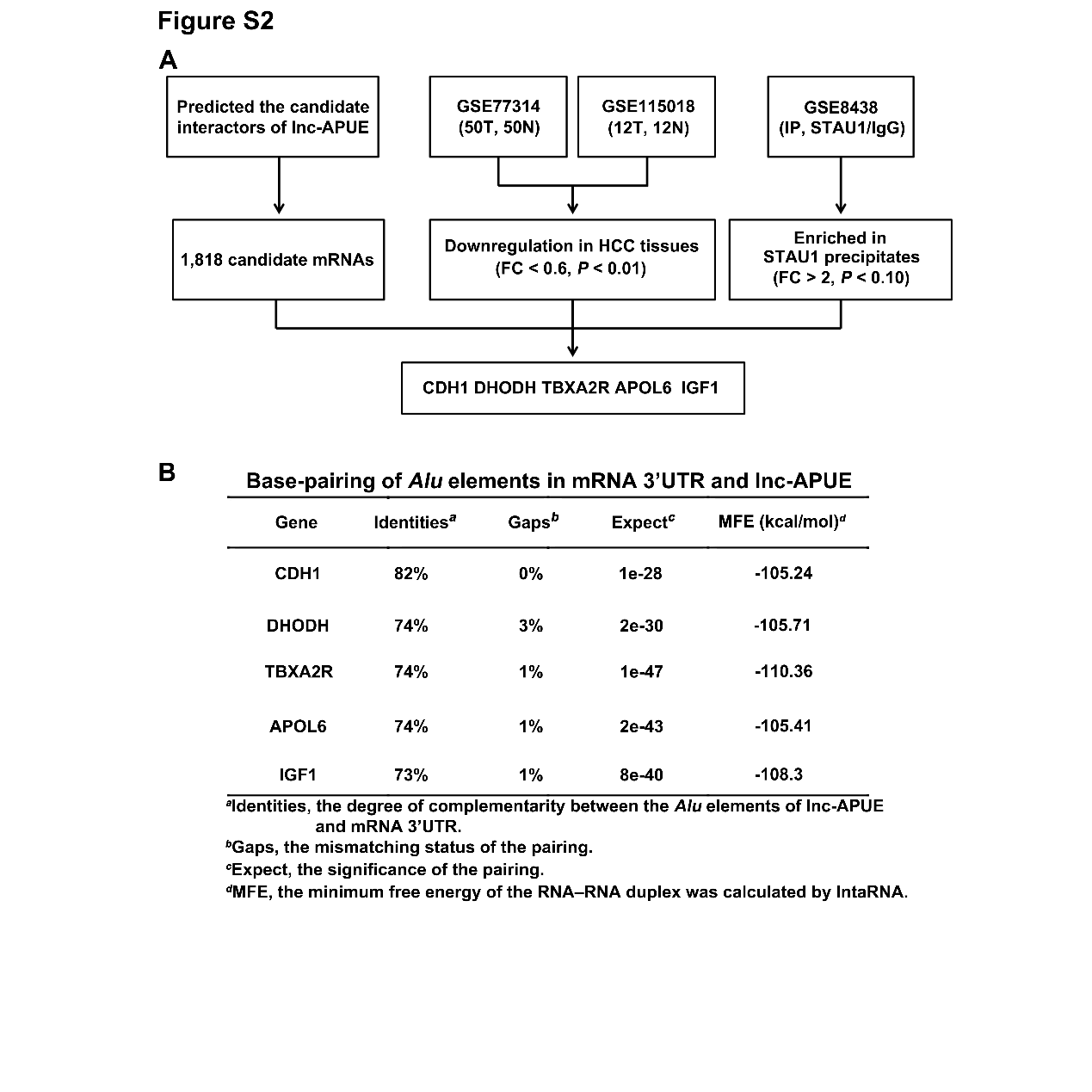


Supplementary Fig. S2. Screening of SMD targets of lnc-APUE during HCC development. (A) The screening workflow for high-confidence HCC-relevant SMD substrates of lnc-APUE. The mRNA expression profiling of paired HCC and noncancerous liver tissues was derived from NCBI Gene Expression Omnibus (GEO, accession numbers are GSE77314 and GSE115018). The levels of STAU1-bound transcripts were from GSE8438. (B) Base-pairings between the *Alu* element in lnc-APUE and the *Alu* element within the 3’UTRs of candidate mRNAs were predicted by IntaRNA and NCBI blastn algorithm.


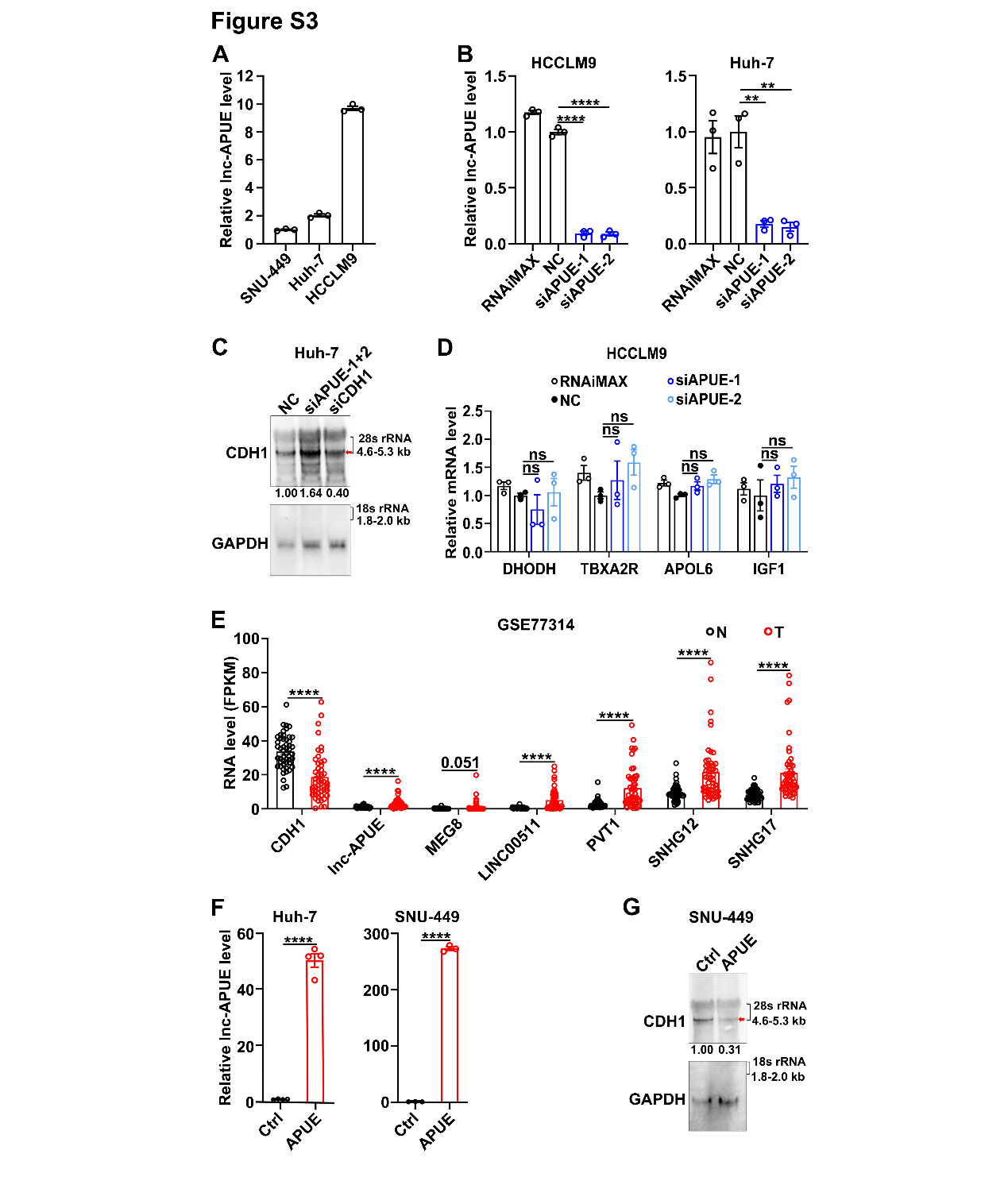


Supplementary Fig. S3. Lnc-APUE reduced CDH1 mRNA levels. (A) Lnc-APUE levels in human hepatoma cell lines. The cellular lnc-APUE in SNU-449, Huh-7 and HCCLM9 was determined by qPCR analysis. (B) The knockdown efficiency of siRNA targeting lnc-APUE. HCCLM9 and Huh-7 cells were transfected with NC, siAPUE-1 or siAPUE-2 for 48 h before qPCR analysis. (C) Northern blotting analysis showed elevation of CDH1 mRNA levels following lnc-APUE knockdown. Huh-7 were transfected with the indicated siRNAs for 48 h before northern blotting analysis. (D) The effects of lnc-APUE knockdown on the mRNA levels of DHODH, TBXA2R, APOL6 and IGF1. HCCLM9 cells were transfected with NC, siAPUE-1 or siAPUE-2 for 48 h prior to qPCR analysis. (E) The expression levels of lnc-APUE, CDH1 and the well-characterized oncogenic lncRNAs. RNA-seq data from 50 paired HCC and adjacent non-tumor tissues (GSE77314) were analyzed. (F) The overexpression efficiency of lnc-APUE. Huh-7 and SNU-449 cells were infected with lentiviruses carrying pCDH-APUE or pCDH-Ctrl, followed by 96-hour culture prior to qPCR analysis. (G) Northern blotting analysis revealed reduction of CDH1 mRNA levels following lnc-APUE overexpression. SNU-APUE and SNU-Ctrl sublines were subjected to northern blotting. For (C, G), red arrow indicates the band of CDH1 mRNA, and the mRNA levels of CDH1 relative to those of GAPDH are indicated under each band. For (B-C, F-G), lnc-APUE is abbreviated as “APUE”. RNAiMAX, cells exposed to Lipofectamine RNAiMAX without RNA. NC, negative control for siRNA. The data from at least three independent experiments are presented as mean ± SEM (A, B, D, F); *P* values were assessed by one-way ANOVA (B, D), paired Student′s *t*-test (E) or unpaired Student′s *t*-test (F). *, *P* < 0.05; **, *P* < 0.01; ****, *P* < 0.0001.


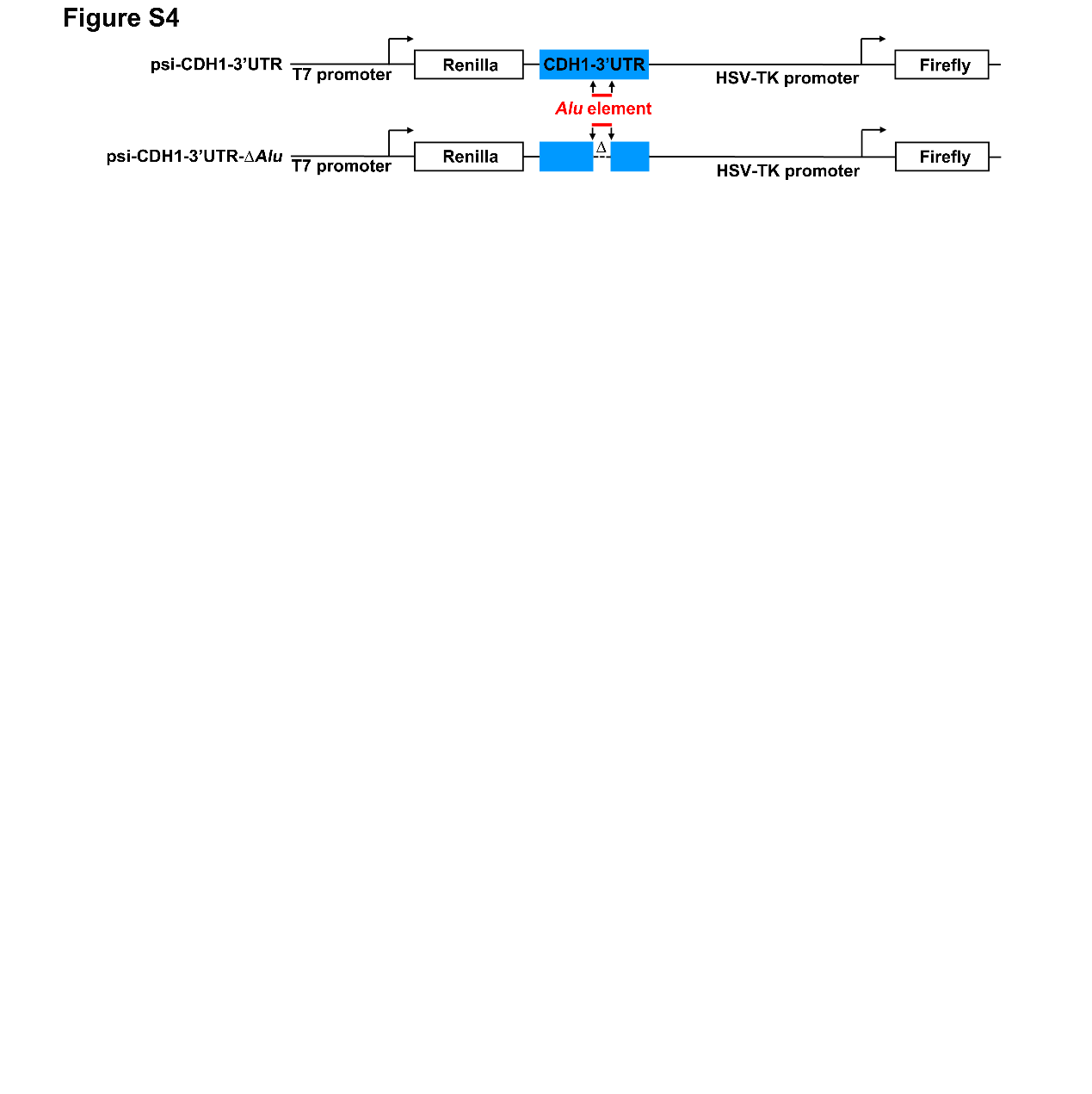


Supplementary Fig. S4. The schematic diagrams of psi-CDH1-3’UTR and psi-CDH1-3’UTR-∆*Alu* constructs. Dotted line in the psi-CDH1-3’UTR diagram denotes the deletion of the *Alu* element base-paired with lnc-APUE.


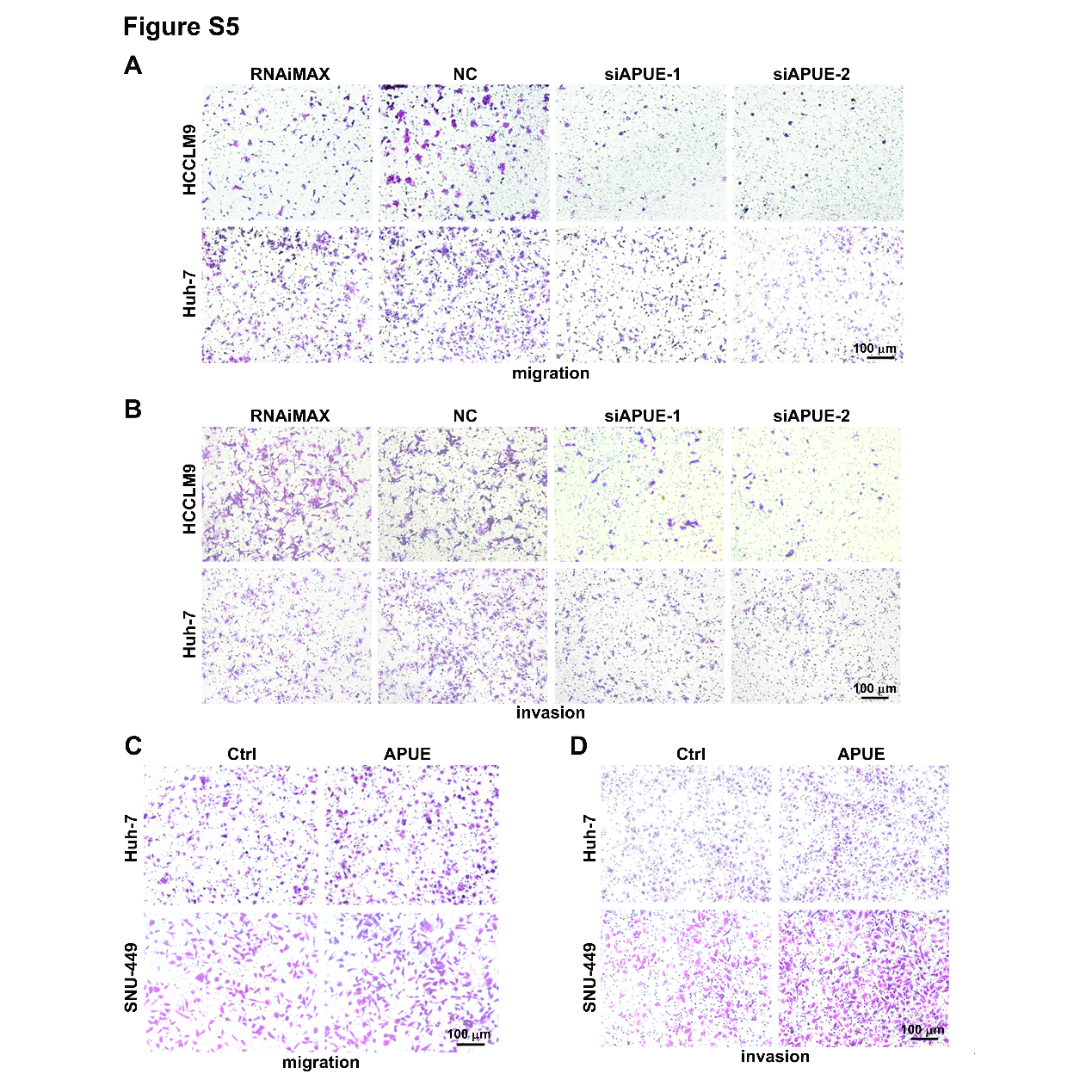


Supplementary Fig. S5. Lnc-APUE promotes the migration and invasion of hepatoma cells. (A-B) Silencing lnc-APUE suppressed the migration and invasion of tumor cells. HCCLM9 and Huh-7 cells were transfected with the indicated RNA duplexes, then cultured for 36 h before migration (A) and invasion (B) analysis. (C-D) Overexpressing lnc-APUE enhanced the migration and invasion of tumor cells. Huh-7 and SNU-449 cells stably expressing lnc-APUE (APUE) and the control cells (Ctrl) were examined. RNAiMAX, cells exposed to Lipofectamine RNAiMAX without RNA. NC, negative control for siRNA. For (A-D), cells were added to transwell chambers without (for migration) or with (for invasion) Matrigel coatings and incubated for 10 h, followed by crystal violet staining. All the migrated/invaded cells were counted. Scale bar, 100 μm. Lnc-APUE is abbreviated as “APUE”.


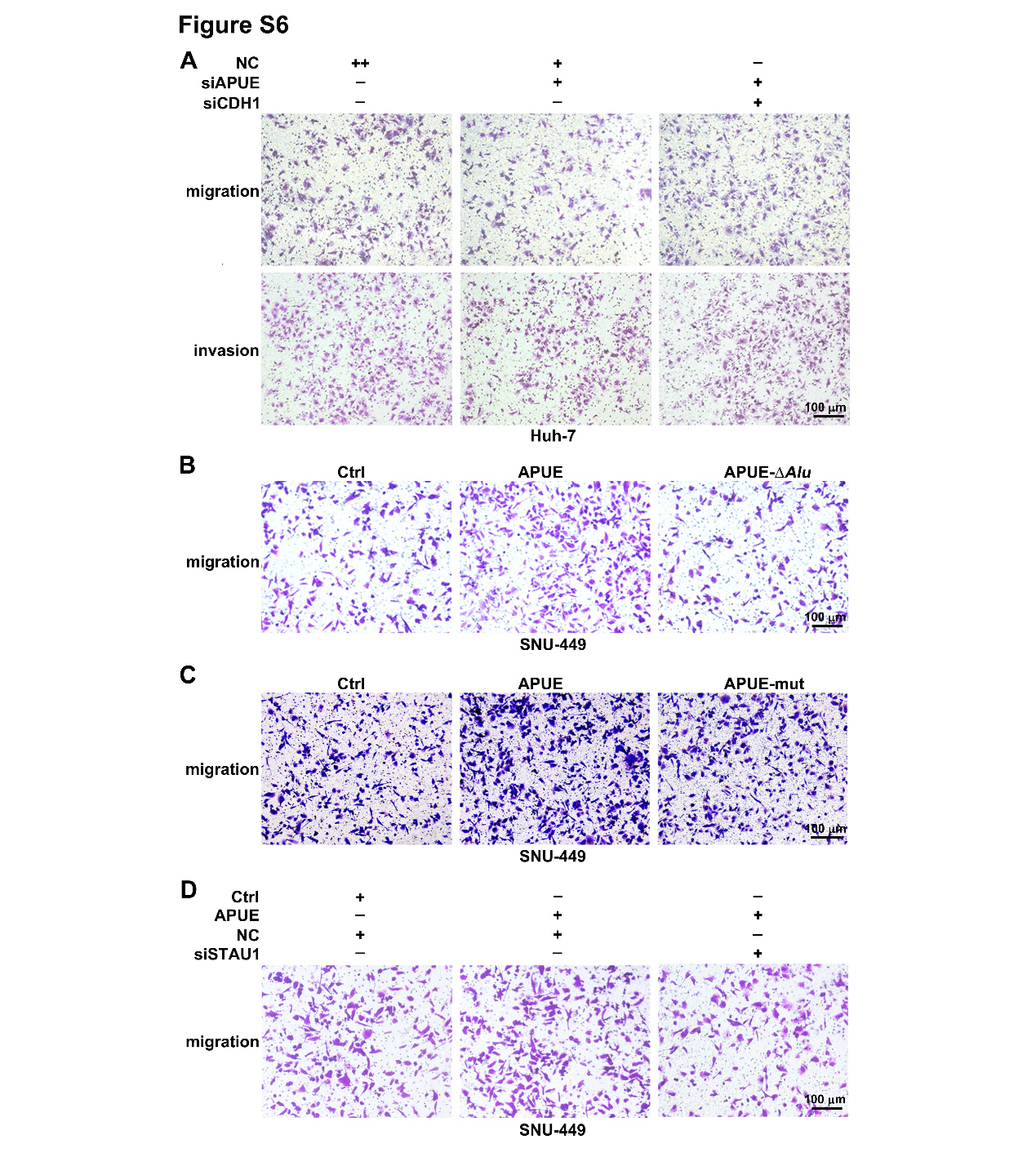


Supplementary Fig. S6. Lnc-APUE promotes migration and invasion of human hepatoma cells by decreasing CDH1 mRNA levels via the SMD pathway. (A) CDH1 silencing abolished the inhibitory effects of lnc‐APUE knockdown on migration and invasion. (B-C) Deletion or mutation of the lnc-APUE *Alu* element abolished the pro-migratory function of lnc-APUE in tumor cells. SNU-449 cells with stable transfection of the control (Ctrl), wild-type (APUE) or *Alu*-deleted (APUE-∆*Alu*) or *Alu*-mutated (APUE-mut) lnc-APUE constructs were analyzed. (D) STAU1 silencing abrogated the pro-migratory role of lnc-APUE in tumor cells. NC, negative control for siRNA. For (A-D), cells were added to transwell chambers without (for migration) or with (for invasion) Matrigel coatings and incubated for 10 h, followed by staining with crystal violet. All the migrated/invaded cells were quantified using a light microscope. Scale bar, 100 μm. Lnc-APUE is abbreviated as “APUE”.


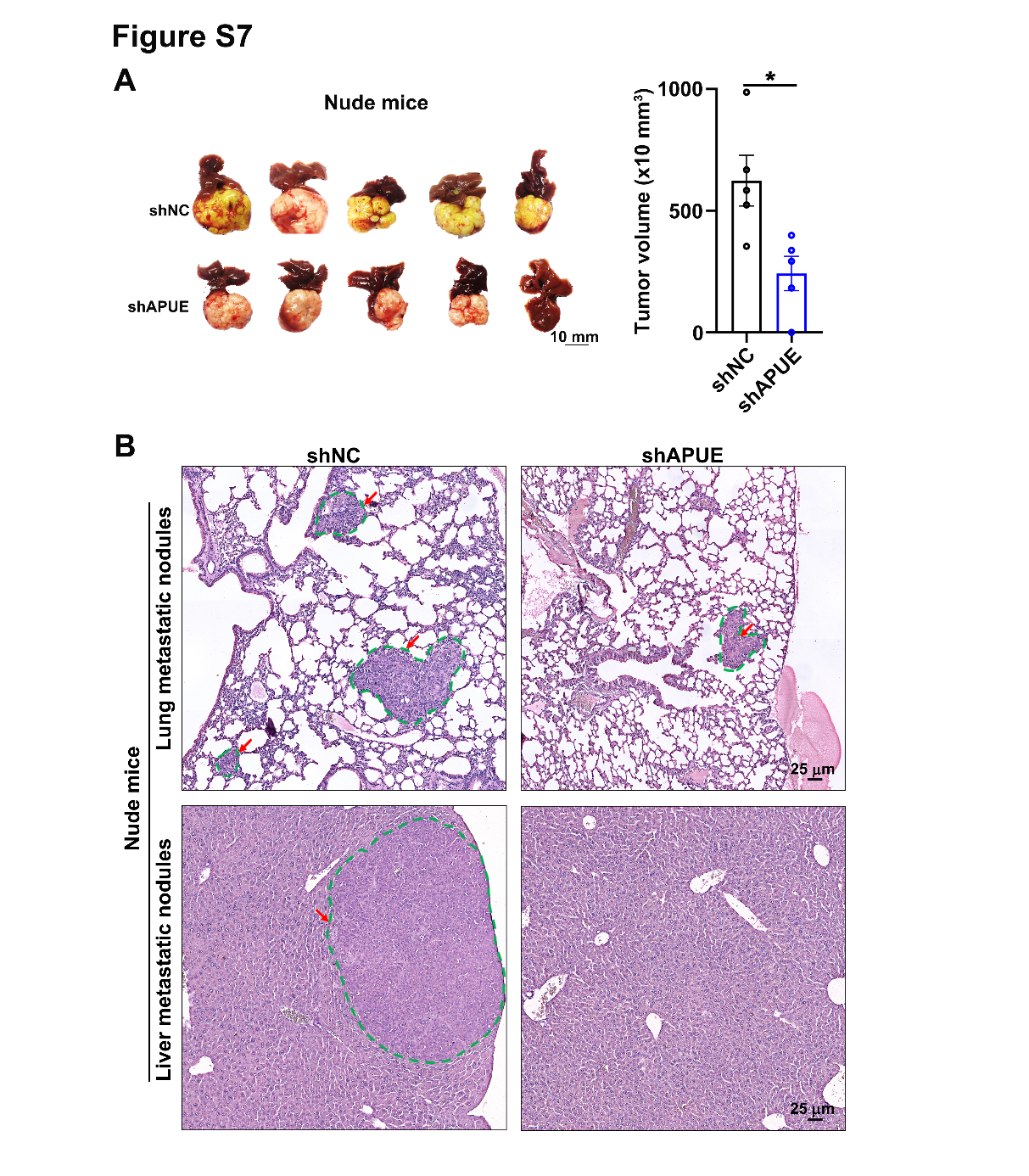


Supplementary Fig. S7. Silencing of lnc-APUE inhibits the growth and metastasis of mouse xenografts. (A) Lnc-APUE knockdown inhibited the growth of hepatoma xenografts in nude mice. LM9-shNC and LM9-shAPUE sublines (n = 5/group) were inoculated under the capsule of the left hepatic lobe of nude mice. Scale bar, 10 mm. (B) The xenografts of lnc-APUE-silenced cells showed fewer and smaller metastatic nodules in the lungs and livers. Representative images of lung and liver metastatic nodules in nude mice. Metastatic nodules in​ the lungs or​ livers are highlighted with green dashed lines and pointed out by red arrows. Scale bar, 25 μm. Lnc-APUE is abbreviated as “APUE”. *P* values were assessed by unpaired Student′s *t*-test (A). *, *P* < 0.05.


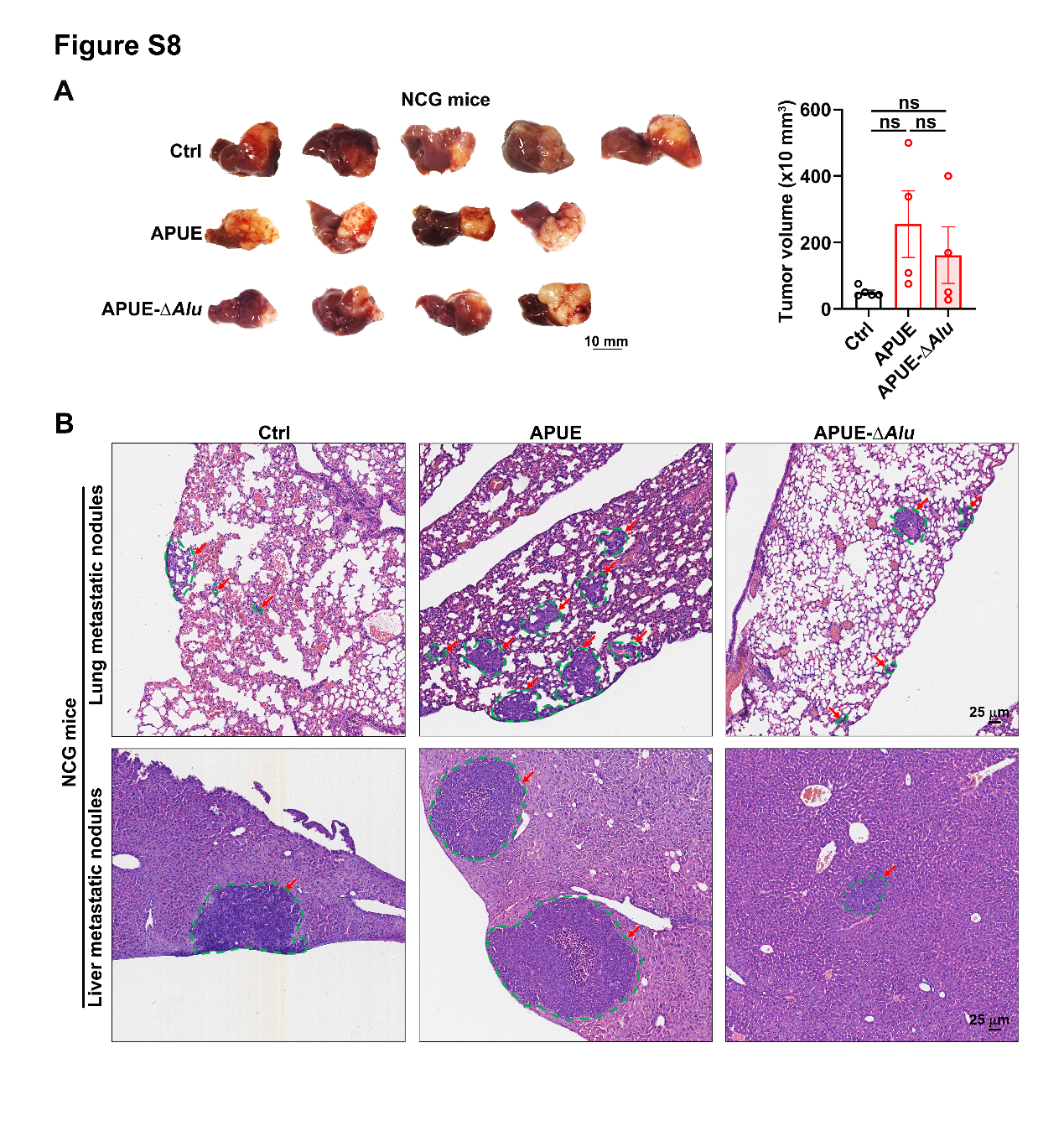


Supplementary Fig. S8. Overexpression of lnc-APUE accelerates tumor growth and metastasis *in vivo*. (A) The promotive effect of lnc-APUE overexpression on xenograft growth in NCG mice. LM9-Ctrl, LM9-APUE and LM9-APUE-∆*Alu* sublines were inoculated under the capsule of the left hepatic lobe of NCG mice (n = 4 - 5/group). Scale bar, 10 mm. (B) Deletion of the *Alu* element in lnc-APUE abolished its function in promoting tumor metastasis *in vivo*. Representative images of lung and liver metastatic nodules in NCG mice. Metastatic nodules in lungs or ​livers are highlighted with green dashed lines and pointed out by red arrows. Scale bar, 25 μm. Lnc-APUE is abbreviated as “APUE”. *P* values were assessed by one-way ANOVA (A). ns, not significant.


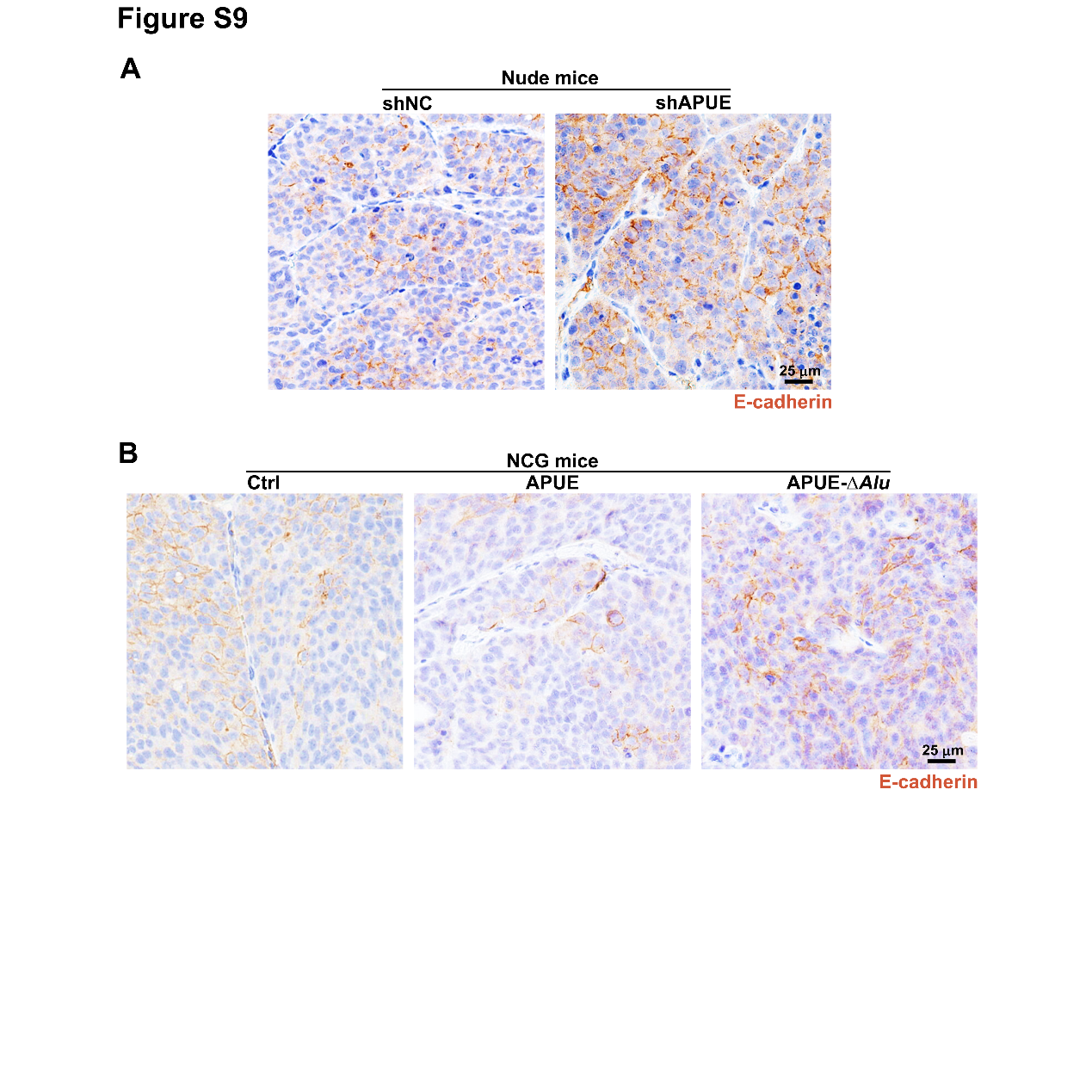


Supplementary Fig. S9. Lnc-APUE reduces E-cadherin protein levels *in vivo* via *Alu* element. (A) Silencing lnc-APUE increased E-cadherin levels in mouse xenografts. (B) Deletion of *Alu* element within lnc-APUE abolished its role in reducing E-cadherin levels. Immunohistochemistry analysis was performed. Scale bar, 25 μm. Lnc-APUE is abbreviated as “APUE”.


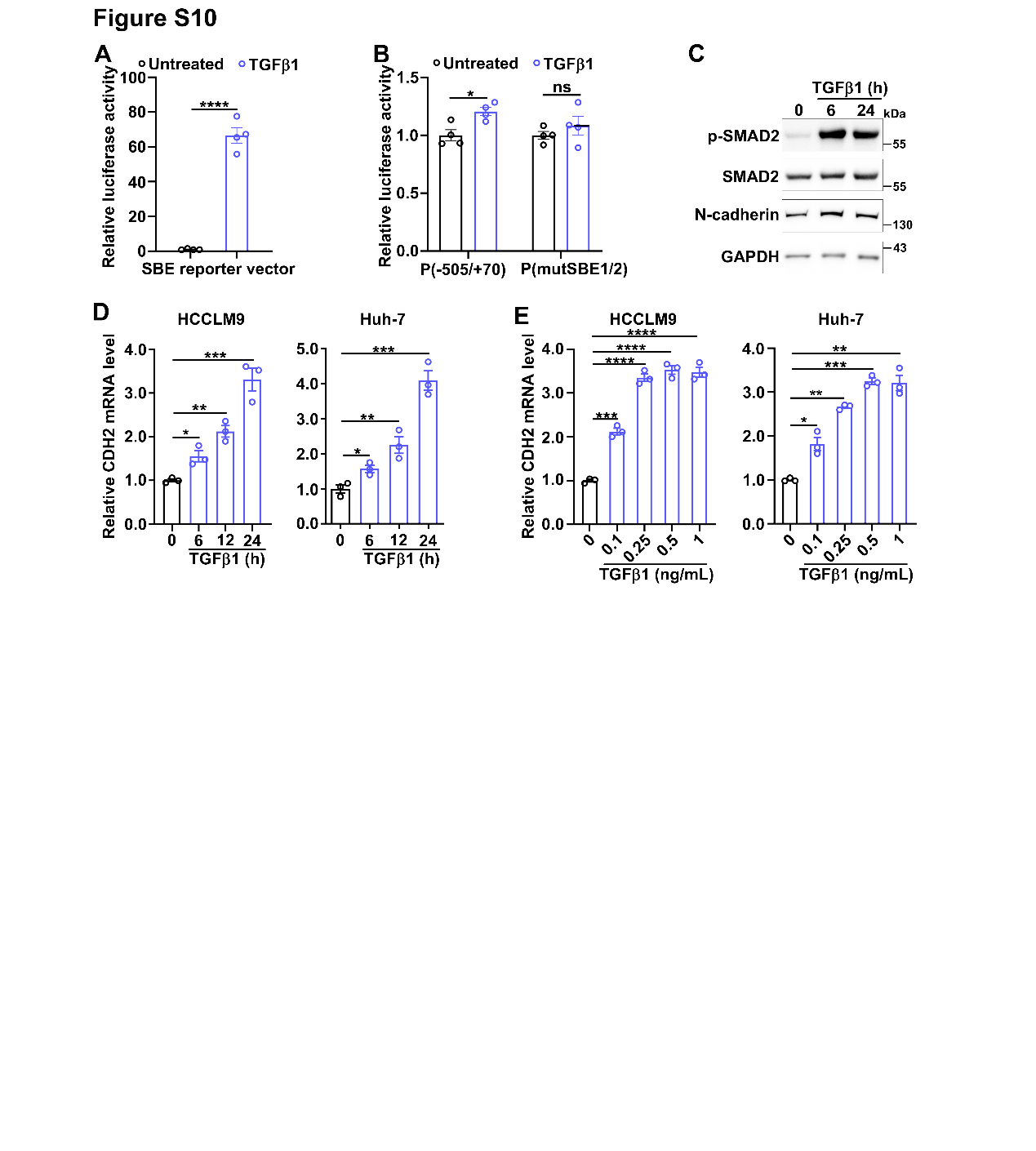


Supplementary Fig. S10. TGFβ1 activates the expression of lnc-APUE and key components of the TGFβ1 pathway. (A) TGFβ1 treatment increased the activity of an SBE reporter. (B) Mutation of putative SBE1 and SBE2 in the lnc-APUE promoter abrogated the response of P(−505/+70 bp) reporter to TGFβ1. For (A-B), HCCLM9 cells were transfected with the indicated vectors for 36 h. The cells were then either left untreated or treated with 1 ng/mL TGFβ1 for a further 12 h prior to the luciferase activity assay. (C) TGFβ1 stimulation increased the levels of phospho-SMAD2 protein and CDH2-encoding N-cadherin protein. Huh-7 cells were untreated or treated with 1 ng/mL TGFβ1 for the indicated time prior to western blotting analysis. (D-E) TGFβ1 treatment significantly upregulated CDH2 in a time- and dose-dependent manner. HCCLM9 and Huh-7 cells were either untreated or treated with 1 ng/mL TGFβ1 for the indicated time (D), or with the indicated dose of TGFβ1 for 24 h (E). Data from at least three independent experiments are presented as mean ± SEM (A-B, D-E); *P* values were assessed by unpaired Student′s *t*-test (A-B) and one-way ANOVA (D-E). *, *P* < 0.05; **, *P* < 0.01; ***, *P* < 0.001; ****, *P* < 0.0001; ns, not significant.


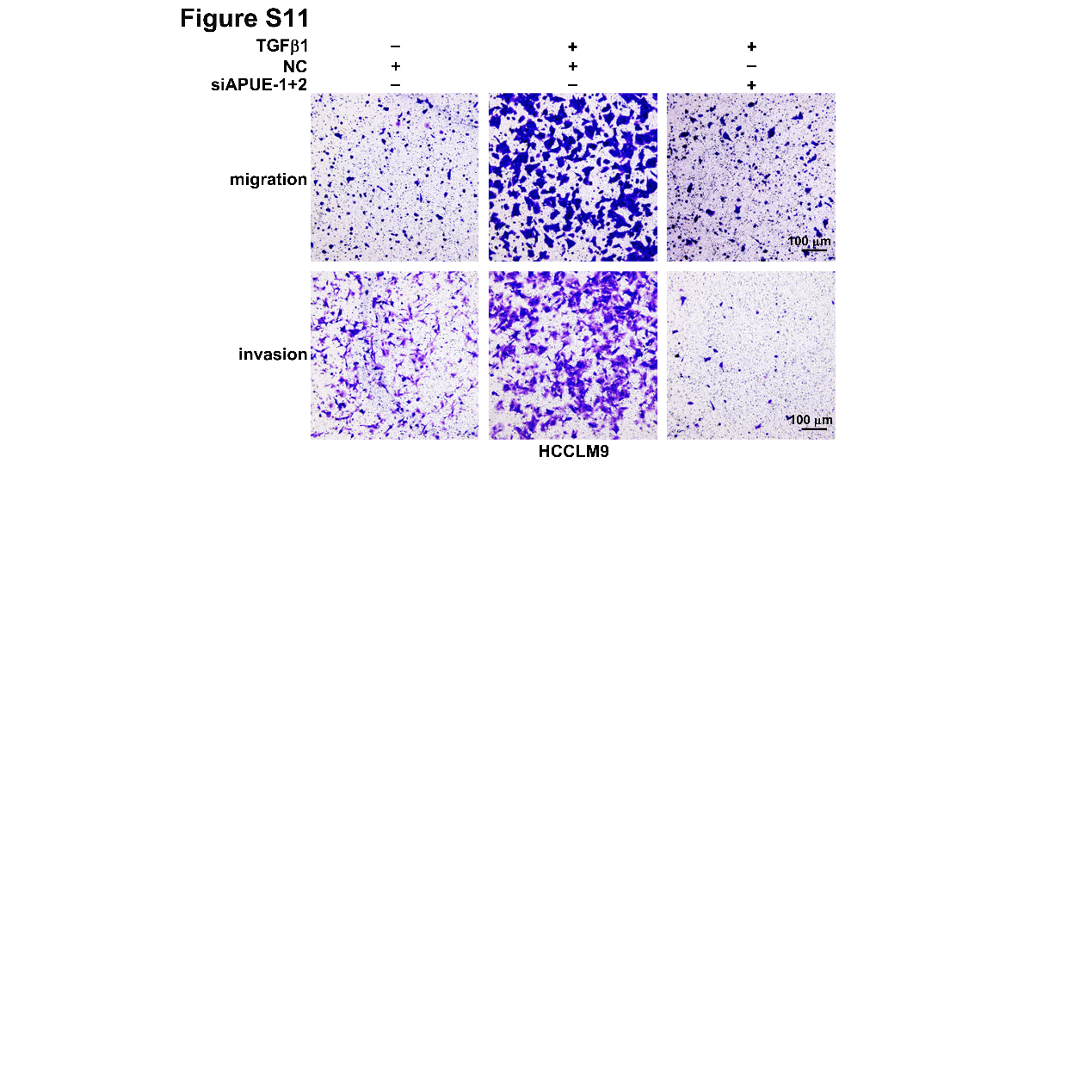


Supplementary Fig. S11. Lnc-APUE silencing abolishes the stimulatory effect of TGFβ1 on tumor cell migration and invasion. HCCLM9 transfected with the indicated RNA duplexes were incubated for 36 h in the absence (-) or presence (+) of TGFβ1, then subjected to migration and invasion assays. NC, negative control for siRNA. Scale bar, 100 μm. Lnc-APUE is abbreviated as “APUE”.


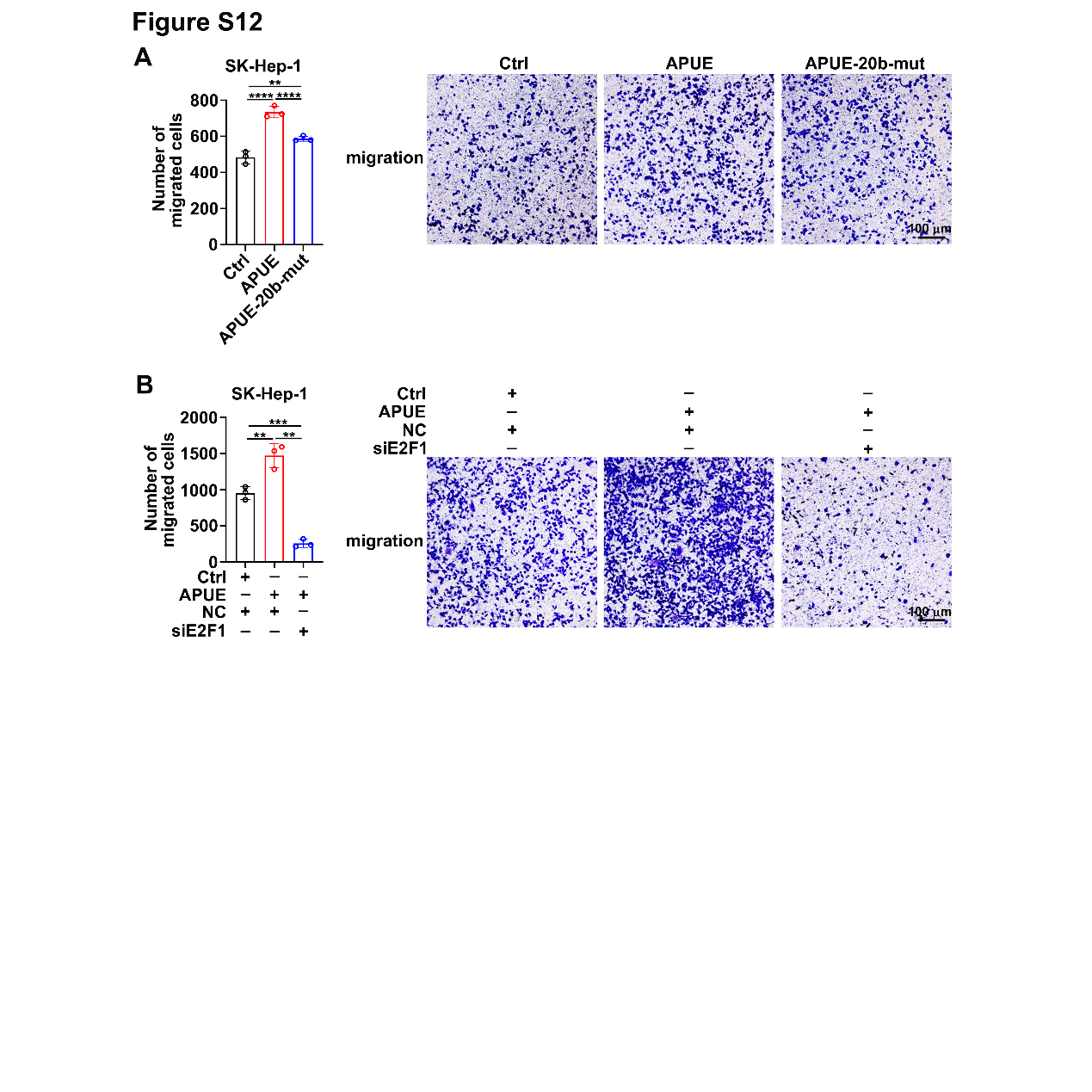


Supplementary Fig. S12. Pro-metastatic capacity of lnc-APUE is attenuated by disrupting lnc-APUE/miR-20b/E2F1 axis. (A) Mutation of the miR-20b-binding sites in lnc-APUE attenuated its ability to promote tumor cell migration. SK-Hep-1 cells stably expressing the control (Ctrl), wild-type (APUE) or miR-20b-binding-site-mutated (APUE-20b-mut) lnc-APUE constructs were analyzed. (B) E2F1 knockdown abrogated the stimulatory effect of lnc-APUE overexpression on tumor cell migration. SK-Ctrl and SK-APUE sublines transfected with the indicated RNA duplexes were subjected to migration assays. NC, negative control for siRNA. Scale bar, 100 μm. Lnc-APUE is abbreviated as “APUE”. Data from at least three independent experiments are presented as mean ± SEM (A-B); *P* values were assessed by one-way ANOVA (A-B). **, *P* < 0.01; ***, *P* < 0.001; ****, *P* < 0.0001.


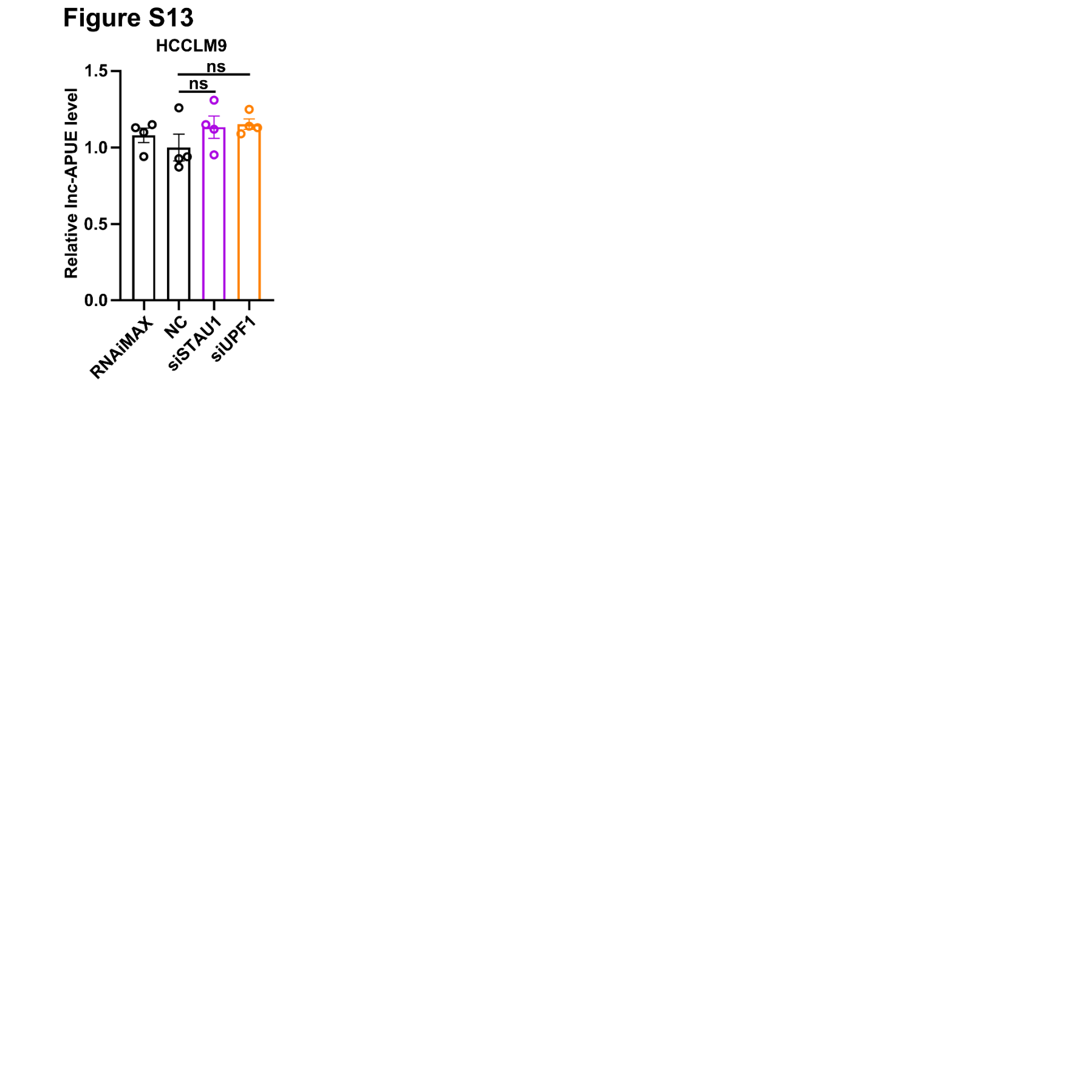


Supplementary Fig. S13. Knockdown of STAU1 or UPF1 does not affect lnc-APUE expression levels. HCCLM9 cells were transfected with indicated siRNAs for 48 h, then subjected to qPCR analysis. RNAiMAX, cells exposed to Lipofectamine RNAiMAX without RNA. NC, negative control for siRNA. The data from at least three independent experiments are presented as mean ± SEM; *P* values were assessed by one-way ANOVA. ns, not significant.

**Supplementary Tables**

**Table S1 Clinical Features of 50 HCC Patients**

| **Variables** | **Cases** | **Variables** | **Cases** |
| --- | --- | --- | --- |
| Gender |  | Tumor size (cm) |  |
| Male | 41 | ＞5 | 38 |
| Female | 9 | ≤5 | 12 |
| Age-yr |  | Tumor number |  |
| ＞50 | 22 | ＞1 | 13 |
| ≤50 | 28 | 1 | 37 |
| HBV |  | PVTT^b^ |  |
| +^a^ | 38 | + | 9 |
| -^a^ | 12 | - | 41 |
| Cirrhosis |  | BCLC stage |  |
| + | 37 | A | 29 |
| - | 13 | B | 5 |
| Ascites |  | C | 16 |
| + | 2 |  |  |
| - | 48 |  |  |
| AFP (ng/ml) |  |  |  |
| ≥400 | 26 |  |  |
| ＜400 | 24 |  |  |

^a^ +, presence; -, absence.

^b^ PVTT, Portal vein tumor thrombus.

**Supplementary Tables**

**Table S2. Sequences of DNA and RNA oligonucleotides**

| **Name** | **Sense strand/sense primer (5′-3′)** | **Antisense strand/antisense primer (5′-3′)** |
| --- | --- | --- |
| **siRNA duplexes** | | |
| siAPUE-1 | GGACUUGAGUGAGAUGUCAdTdT | UGACAUCUCACUCAAGUCCdTdT |
| siAPUE-2 | GGGAAGACAGCCAGGUGAAdTdT | UUCACCUGGCUGUCUUCCCdTdA |
| siCDH1-1 | CAGACAAAGACCAGGACUAdTdT | UAGUCCUGGUCUUUGUCUGdAdC |
| siCDH1-2 | GGGACAACGUUUAUUACUAdTdT | UAGUAAUAAACGUUGUCCCdGdG |
| siSTAU1-1 | CCUAUAACUACAACAUGAGdTdT | CUCAUGUUGUAGUUAUAGGdTdG |
| siSTAU1-2 | GUUUGAGAUUGCACUUAAAdTdT | UUUAAGUGCAAUCUCAAACdAdC |
| NC | UGAAUUAGAUGGCGAUGUUdTdT | AACAUCGCCAUCUAAUUCAdTdT |
| siUPF1 | GAUGCAGUUCCGCUCCAUUdTdT | AAUGGAGCGGAACUGCAUCdTdT |
| siSMAD2-1 | GAAUUGAGCCACAGAGUAAdTdT | UUACUCUGUGGCUCAAUUCdTdT |
| siSMAD2-2 | GCCCUCACUCACUGUAGAUdTdT | AUCUACAGUGAGUGAGGGCdTdT |
| siSMAD3-1 | CGUCAACACCAAGUGCAUCdTdT | GAUGCACUUGGUGUUGACGUU |
| siSMAD3-2 | CCGCAUGAGCUUCGUCAAAdTdT | UUUGACGAAGCUCAUGCGGdAdT |
| **Name** | **Sense strand/sense primer (5′-3′)** | **Antisense strand/antisense primer (5′-3′)** |
| siSMAD4-1 | GCCAGCUACUUACCAUCAUdTdT | AUGAUGGUAAGUAGCUGGCdTdG |
| siSMAD4-2 | GCCUCCCAUUUCCAAUCAUdTdT | AUGAUUGGAAAUGGGAGGCdTdG |
| siTGFBR1-1 | GGAGAUUGUUGGUACCCAAdTdT | UUGGGUACCAACAAUCUCCdAdT |
| siTGFBR1-2 | CCAUUGAUAUUGCUCCAAAdTdT | UUUGGAGCAAUAUCAAUGGdTdA |
| siE2F1 CACUGAAUCUGACCACCAAdTdT UUGGUGGUCAGAUUCAGUGdAdG | | |
|  | | |
| **Primers for qPCR** | | |
| APUE | GGGTGGATGCTGTCAGACTT | ACCGGGTCTGGCTATATTGC |
| CDH1 | GCCCCATCAGGCCTCCGTTT | ACCTTGCCTTCTTTGTCTTTGTTGGA |
| CDH1 3’UTR | CTTGGAGATGGCAGGAGAGC | TGGGGATTCTGGGCTTTGAG |
| GAPDH | AGAAGGCTGGGGCTCATTTG | AGGGGCCATCCACAGTCTTC |
|  | | |
| **Probes for northern blotting** | | |
| CDH1 CAAGTCAAAGTCCTGGTCCTCTTCTCCGCCTCCTTCTTCATCATAGTAAT | | |
| GAPDH GGGTGGCAGTGATGGCATGGACTGTGGTCATGAGTCCTTCCACGATACCA | | |
| **Name Sense strand/sense primer (5′-3′) Antisense strand/antisense primer (5′-3′)** | | |
| **Probes for RNA-RNA EMSA**  CDH1-*Alu*  AUUUUUUUGUACAGAUGGGGUCUUGCUAUGUUGCCCAAGCUGGUCUUAAACUCCUGGCCUCAAGCAAUCCUUCUGCCUUGGCCCCCCAAAGUGCUGGGAUUGUGGGCAUGAGC  APUE-*Alu*-wt GCUCACACCUAUAAUCCCAGCACUUUGGGAGGCUGAGGCACGAGGAUUGCUUGAGCCCAGGAGUUCAUCACCAGCCUGGGCAAUAUAGCCAGACCCGGUCUCCACAAAAAAAU  APUE-*Alu*-mut CGAGUGUGGAUAUUAGGGUCGUGAAACCCUCCGACUCCGUGCUCCUAACGAACUCGGGUCCUCAAGUAGUGGUCGGACCCGUUAUAUCGGUCUGGGCCAGAGGUGUUUUUUUA | | |
|  | | |
| **Primers for pCDH vector cloning** | | |
| APUE | GCTCTAGAGCTGGGGTCATTGGGCTGCCTT | GGAATTCCCCCAGTCTCACAGGGGCCCAT |
| STAU1 | GCTCTAGAGCATGTCTCAAGTTCAAGTGC | ATAAGAATGCGGCCGCTAAACTATCT  TATCGTCGTCATCCTTGTAATCGCACCTC  CCACACAC |
| **Name** | **Sense strand/sense primer (5′-3′)** | **Antisense strand/antisense primer (5′-3′)** |
| S1m-APUE | GGAATTCCTGGGGTCATTGGGCTGCCTT | CGCATTTAAATCCCAGTCTCACAGGGGCCCAT |
| S1m-APUE-Δ*Alu* | GCAGGGCCAACCTGTGCCTGTAGTCCCAGCT | TACAGGCACAGGTTGGCCCTGCCACATGCCA |
|  |  |  |
| **Primers for pGL3-basic vector cloning** | | |
| P(-1553/+70bp) | GGGGTACCCCGCATCAGGTTCAAAGCCAGG | CCCAAGCTTGGGTGCAGAGCTTTGTCC  AGAGG |
| P(-889/+70bp) | GGGGTACCCCAGATCGCACCATGGCACT |  |
| P(-505/+70bp) | GGGGTACCCCGTCTTGTTGGGACTCTCGGG |  |
| P(-212/+70bp) | GGGGTACCCCCCCTGCTCCCGGTGAAT |  |
| P(del SBE1/2) | GGGGTACCCCTGTTGGGACTCTCGG  GTGCACTGAGCACACTTCCTC |  |
| P(del SBE3) | GGCCTCTGCATCCAGGGGCACCTGTG | TGCCCCTGGATGCAGAGGCCCGGGGCA |
| P(mutSBE1/2) | CCGCTCGAGCGGGACATGTTGGGACTCTCGGGGACATGCACTGAGCACACTT | CCCAAGCTTGGGTGCAGAGCTTTGTCCAGAGG |
|  |  |  |
| **Primers for psiCHECK2 vector cloning** | | |
| **Name Sense strand/sense primer (5′-3′)**  **Antisense strand/antisense primer (5′-3′)** | | |
| CDH1-3’UTR | AGTCTCGAGTCTCATTCTATCGGCCAGGC | AGTGCGGCCGCCCACCTCAATCATCCTCAGCA |
| CDH1-3’UTR-Δ*Alu* | AAGCAGTTCTCTGCTGTGCCCAGCCTCCATG | GGGCACAGCAGAGAACTGCTTGAGCCCCA |
|  |  |  |
| **Primers for ChIP-PCR** | |  |
| CDH2 | AGTACATCCTCAAGGGTGGG | TCATTCTTTGGAGATGGGTA |
| Primer 1 | CAGGCTTAGCCAAACCCTAA | AAAGTGCTGCTGGGATTTCA |
| Primer 2 | GGCACCTGTAATCCCAGCTA | GGAGTTTCCCTCTGTTTCCC |
| Primer 3 | GGCTAGCTCACCCTCCTAAA | GCTCCTACTTTCCTGCCAGA |
| Primer 4 | TTTCCCAGTCCAAAGGTCAC | CAAGCTCAGCCTCTTCCTGT |
| GAPDH | TACTAGCGGTTTTACGGGCGCACGT | AACAGGAGGAGCAGAGAGCGAA |
